# Resistance breaking Root-knot nematodes carry a fitness cost associated with defective feeding site development

**DOI:** 10.1371/journal.ppat.1014581

**Published:** 2026-09-11

**Authors:** Alison C. Blundell, Emma Shigekane-Kraft, Sławomir Janakowski, Mirosław Sobczak, Dadong Dai, Yali Zhang, Pallavi Shakya, Ching-Jung Lin, Valerie M. Williamson, Shahid Siddique

**Affiliations:** 1 Department of Plant Pathology, University of California, Davis, Davis, California, United States of America; 2 Department of Entomology and Nematology, University of California, Davis, Davis, California, United States of America; 3 Department of Botany and Plant Physiology, Institute of Biology, Warsaw University of Life Sciences, Warsaw, Poland; University of California Riverside, UNITED STATES OF AMERICA

## Abstract

Root-knot nematodes (RKNs) cause an estimated 157 billion dollars in annual yield losses worldwide. In tomato, resistance to RKNs is conferred by the single dominant resistance gene *Mi-1*, which has been widely integrated into commercial cultivars. Prolonged and widespread use of *Mi-1* has led to the emergence of resistance-breaking populations in tomato fields worldwide; however, the consequences of resistance breaking for nematode performance on susceptible hosts remain poorly understood. Here, we compared infection outcomes of two closely related strains of *Meloidogyne javanica,* VW4 (*Mi-1*–avirulent; wild-type) and VW5 (*Mi-1*–virulent; resistance-breaking), on three susceptible hosts: tomato, cucumber, and rice. Across all hosts, VW5 produced significantly fewer eggs than VW4, revealing a fitness cost associated with *Mi-1* virulence. Light and transmission electron microscopy of tomato and cucumber galls revealed impaired feeding site establishment by VW5. Consistent with these observations, transcriptomic profiling of nematode-infected roots showed that VW5 infection induced weaker host transcriptional reprogramming than VW4 and lacked host gene expression signatures associated with effective suppression of plant defense responses. Together, these findings demonstrate that adaptation to *Mi-1*-mediated resistance is accompanied by a fitness cost on susceptible plants and impaired feeding site formation and altered host reprogramming. Furthermore, these results establish VW4 and VW5 as a powerful resource for understanding host genes and pathways required for successful parasitism and feeding site development.

## Author summary

Root-knot nematodes are microscopic plant parasites that cause major yield losses in many crops, including tomato, cucumber, and rice. Tomato cultivars that carry the resistance gene *Mi-1* can prevent successful parasitism by several root-knot nematode species. However, some nematode populations have evolved the ability to overcome this resistance, compromising the durability of resistant cultivars. In this study, we compared two closely related strains of *Meloidogyne javanica*: VW4, which cannot reproduce on tomato with the *Mi-1* resistance gene, and VW5, which successfully reproduced in resistant tomato. However, VW5 produced substantially fewer eggs than VW4 on susceptible tomato, cucumber, and rice, indicating a reproductive fitness cost associated with breaking resistance. Microscopy revealed that VW5 induced abnormal and structurally compromised feeding sites in susceptible plants. Comparison of cucumber gene expression in infected roots showed that VW5 caused weaker early reprogramming of host gene expression than did VW4, particularly in pathways associated with feeding-site development and host-cell remodeling. Together, these findings indicate that the ability to overcome *Mi-1* resistance is associated with reduced performance on susceptible hosts due to impaired ability to manipulate host tissues.

## Introduction

Plant-parasitic nematodes (PPNs) cause an estimated 157 billion U.S. dollars in annual crop losses worldwide [1]. Root-knot nematodes (RKNs; *Meloidogyne* spp.) are the most destructive group of PPNs and infect more than 3,000 plant species, including tomato, cucumber, soybean, and rice [2–4].

RKNs begin their life cycle as motile second-stage juveniles (J2s) that typically penetrate roots near the root tip, particularly within the elongation zone, and migrate toward the vascular cylinder. When J2s enter more basal regions of the root, they first migrate toward the root tip before turning toward the vascular cylinder. Upon reaching the vascular tissues, nematodes become sedentary and establish permanent feeding sites composed of modified host cells known as giant cells (GCs) [5,6]. These GCs function as hypermetabolic sinks, and once established, nematodes remain sedentary and depend exclusively on them for nutrient acquisition throughout the remainder of their life cycle [5]. During feeding site initiation, maintenance, and development, RKNs extensively reprogram host cells to support GC formation and function. Typically, five to seven GCs are induced per nematode and are characterized by cellular and nuclear hypertrophy, dense cytoplasm, abundance of organelles, multinucleation, and an absence of a large central vacuole. These cells also develop extensive systems of cell wall ingrowths on walls facing newly differentiated xylem vessels, thus facilitating nutrient transport to the feeding site and nematode [7]. Without functional giant cells, nematodes are unable to feed and ultimately die [8].

The tomato resistance gene *Mi-1* (also referred to as *Mi-1.2*) has been incorporated into numerous tomato cultivars worldwide to manage RKNs. In some production systems, including processing tomatoes, it is estimated that more than 95% of commercial cultivars carry *Mi-1*. *Mi-1* confers resistance against several RKN species, including members of the *Meloidogyne incognita* group (*M. incognita, M. javanica,* and *M. arenaria*) and has also been reported to be effective against *M. luci* and *M. ethiopica* [9–11]. Notably, *Mi-1* does not prevent initial nematode penetration: J2s still enter roots and attempt to establish feeding sites. However, shortly after initial feeding cell selection, targeted host cells collapse, and a hypersensitive response (HR) is triggered at the attempted feeding site. HR can be detected as early as 12 hours post-infection, and nematodes either die from starvation or leave the root [12,13]. Despite its efficacy, overreliance on *Mi-1* has resulted in the emergence of resistance-breaking RKN populations in tomato fields worldwide [14].

Previous studies have shown that resistance-breaking in plant parasitic nematodes can be associated with fitness costs on susceptible hosts. For example, reduced reproduction has been reported for resistance breaking strains of *M. incognita* on susceptible tomato and in some cases on pepper [14–16]. However, these studies generally lacked direct progenitors of the resistance breaking strains, making it difficult to determine whether reduced fitness reflected a true cost of resistance or simply differences in baseline virulence among genetically distinct populations.

Two closely related strains of *M. javanica,* VW4 (*Mi-1*–avirulent and unable to reproduce on *Mi-1* tomato) and VW5 (derived from VW4 and *Mi-1*–virulent, capable of reproducing on resistant tomato) have been used as a model system to investigate mechanisms underlying *Mi-1* resistance-breaking [17] (**S1 Fig**). Since VW5 was derived from a greenhouse culture of VW4 after only two generations of selection on resistant tomato, this strain pair provides an opportunity to directly assess fitness costs associated with *Mi-1* virulence while minimizing confounding effects of unrelated genetic variation. As such, differences between VW4 and VW5 can be attributed more confidently to traits associated with *Mi-*1 virulence rather than inherent differences in overall pathogenicity.

In this study, we examined host responses and feeding site development using RNA-seq together with light and transmission electron microscopy of galled tissues. Our analyses show that VW5 is less fit on susceptible hosts and displays weaker host reprogramming leading to underdeveloped feeding sites, consistent with a reproduction cost. Notably, this effect differed significantly with host. Together, these findings demonstrate that comparing VW4 and VW5 provides a useful approach to identify mechanisms required for successful nematode infection and feeding site development as well as clues to loss and gain of host range by these asexually-reproducing species.

## Methods

### Nematode inoculation and maintenance

*Meloidogyne javanica* populations were maintained for 3 months on nematode-resistant *Solanum lycopersicum* resistant (cv. VFNT) and susceptible (cv. Momar Verte) plants [18]. Seeds were germinated and transplanted under greenhouse conditions (26°C, nutrient irrigation via drainage 3 times a day for 3 minutes each) in 1-liter pots with sterile sand. To extract nematode eggs, roots were rinsed to remove sand and cut into ~1 cm-long segments. Root segments were shaken for 3 minutes in 10% [v/v] bleach (6% NaClO) and rinsed thoroughly with water over sieves (75 µm and 25 µm subsequently) until residual bleach was removed. Eggs were collected from the lower sieve into a 50 mL Falcon Tube. Following this, eggs were surface sterilized and set up for hatching as described in Liu and Williamson [19]. For infection assays, hatched J2s were counted and adjusted to 500 J2s/mL and each infected plant received 1 mL inoculum. All assays consisted of three independent experiments.

### Plant material, germination, and growth

Experimental plant species included tomato (*S. lycopersicum* cv. Moneymaker and resistant cv. Motelle), cucumber (*Cucumis sativus* cv. Marketmore76), and rice (*Oryza sativa* cv. Kitaake). Seeds were surface sterilized prior to infection assays as described in Yimer et. al. [20]. Sterile tomato and rice seeds were germinated on moist filter paper in darkness at 28°C for 5–7 days, whereas cucumber seeds were directly planted into pots. Plants used for greenhouse assays were grown in 1-liter Styrofoam pots filled with sterile sand under the same conditions as described above for nematode culture maintenance.

### Nematode infection assays

Seedlings (three weeks old) were inoculated with 1 mL of inoculum containing 500 J2s/mL of either VW4 or VW5 J2s. For each assay, three independent experimental replicates were conducted. For nematode egg collections, roots were harvested at 35 days post inoculation (dpi) and aboveground tissues were discarded. Fresh root weight was recorded in grams. For infection assays, roots were not cut prior to egg collection to allow subsequent staining when required. Collected eggs were counted for all plant cultivars tested. For tomato, stained roots were also used to count the numbers of females and galls at 35 dpi.

### Root staining and nematode visualization

Following egg collection for infection assays at 35 dpi, intact roots were stained in Acid Fuchsin (ThermoFisher Scientific, Waltham, MA, USA: 227905000). Roots were immersed in boiling stain for three minutes for infection assays and one minute for invasion assays with constant agitation, rinsed thoroughly with water, and stored in glycerol until observation.

### Quantification of nematode life stages and galls

Nematodes and galls were counted using a Leica S8 APO dissecting microscope (Leica Microsystems, Wetzlar, Germany). Each nematode was counted as a single individual. Galls were dissected as needed to ensure accurate nematode counts. Each data point represents counts per individual plant.

### Attraction assays

Attraction of J2s to roots was assessed using a Pluronic gel assay (Wang et al.) Seeds for tomato (cv. Moneymaker), cucumber (cv. Marketmore76), and rice (cv. Kitaake) were surface sterilized and germinated on moist filter paper at 28°C for 7 days as described above. Using a 6-well cell culture plate, 2 mL of 23% Pluronic gel were added to each well. After germination of seedlings, a 1 cm root tip was cut and placed on the Pluronic gel for each plant variety. Sterile J2s (200 J2s) for either VW4 or VW5 were added to each well 5 mm from the root tip. The number of J2s touching the root tip were recorded at 30 minutes, 1 hour, and 2 hours. Each assay had 12 replicates per host plant, and three sets of experiments were conducted. Outliers were removed and Line graphs were generated using GraphPad Prism.

### Sampling of galled tissues for microscopy

Susceptible (cv. Moneymaker) and resistant (cv. Motelle) tomato along with cucumber (cv. Marketmore76) seedlings (three-weeks old) were inoculated with J2s of *M. javanica* as described above. Collected samples consisted of galled root segments on infected plants and roots segments from uninoculated plants/roots at comparable locations. Galls and uninfected root segments were collected at 7 and 28 dpi. Root and galled segments were immediately transferred into a modified Karnovsky’s fixative solution composed of 2% [w/v] paraformaldehyde and 2% [v/v] glutaraldehyde dissolved in 50 mM sodium cacodylate buffer, pH = 7.2. The samples were incubated for 2 hours and thereafter the fixative was replaced with 50 mM cacodylic buffer (pH = 7.2) and incubated for 10 mins. The washing steps were repeated 4 times, and the samples were stored in the last buffer bath until further processing.

### Gall sectioning and imaging

The samples were post-fixed in a 2% [w/v] aqueous solution of osmium tetroxide (OsO_4_) at room temperature in darkness for 2 hours and rinsed with cacodylic buffer as described above. Following this, samples were dehydrated in a graded series of ethanol solutions (10% [v/v] increments) for 15 minutes each. The final dehydration was performed with pure ethanol (with 3 baths over 60 minutes in total). Ethanol was then substituted with pure propylene oxide (3 baths for 20 minutes each) before sample infiltration with mixtures (4:1, 2:1, 1:1, 1:2, 1:3; 1:4 [v/v]) of propylene oxide and EPON-type epoxy resin (Agar Scientific, Stansted, UK) for 2 hours each. Samples were then incubated in pure resin for 24 hours (replacing resin every 6 hours). After this, the samples were transferred into flat embedding molds filled with pure resin and positioned for transverse sectioning. Finally, the resin was cured at 65 °C for 12 hours.

Serial sections for light microscopy examination (3 µm thickness) were produced on glass knives using a Leica RM2165 microtome (Leica Microsystems). Sections were stained in 0.1% [w/v] aqueous solution of Toluidine Blue in 50 mM phosphate buffer (pH = 6.9) at 65 °C for 3 minutes. They were examined under an Olympus AX70 ‘Provis’ light microscope (Olympus, Tokyo, Japan) in the bright-field mode. Digital images were taken with an Olympus UC90 digital camera (Olympus). Images were resized, cropped and equalized for similar contrast and brightness using Adobe Photoshop image processing software (Adobe Inc., San Jose, CA, USA).

Sections for transmission electron microscopy were taken at defined places based on analyses of light microscopy images. Ultra-thin (80 nm thickness) sections were cut on a Leica Ultracut E ultramicrotome (Leica) equipped with a diamond knife (DiATOME, Nidau, Switzerland). The sections were collected on formvar coated, single slot copper grids (Agar). Prior to examinations sections were counter-stained in saturated solution of uranyl acetate in 70% [v/v] methanol and 2.5% [w/v] aqueous solution of lead citrate. After air-drying the sections were examined under a FEI268d ‘Morgagni’ transmission electron microscope (FEI Comp., Hillsboro, OR, USA) operating at 80 kV and equipped with a SIS-Olympus ‘Morada’ digital camera (Olympus) with 16 MPix resolution. Digital images were resized and equalized for similar contrast and brightness using Adobe Photoshop.

### Infection assays for transcriptional analysis

Plants were grown under controlled growth room conditions as described above and infected at three-weeks old. Galls on infected plants and uninoculated roots on uninfected plants were collected at 7 and 28 dpi following infection by VW4 or VW5. Upon dissection, excised galls or healthy tissues were flash frozen in liquid nitrogen. Samples were randomly pooled into three sample batches and stored in the -80°C until RNA extraction.

### RNA extraction and sequencing

RNA was extracted using the RNeasy Mini Kit (QIAGEN, Hilden, Germany: 74106). Samples were ground using metal beads and a tissue homogenizer. RNA concentrations were measured using a NanoDrop spectrophotometer (ThermoFisher Scientific: 13-400-526) and adjusted to needed values for sequencing. RNA samples were submitted to Novogene (Novogene Corporation Inc., Sacramento, CA, USA). Samples were checked for quality and ran on a NovaSeq X Plus 25B instrument (Novogene) and complied into a mRNA library preparation (poly A enrichment) using the ABClonal mRNA-Seq Library Prep Kit for Illumina (Catalog: RK20302).

### RNAseq processing differential expression analysis

The raw data was filtered and trimmed to obtain clean reads [21]. The cleaned reads were then mapped to the cucumber reference genome Chinese long version 4 (Clv4) [22] using HISAT2 [23]. Differentially expression genes (DEGs) analyses were performed using DESeq2 [24]. Raw read counts were modeled using a negative binomial distribution in DESeq2, and differential expression was assessed using the Wald test. p-values were adjusted for multiple testing using the Benjamini-Hochberg procedure. Genes with an adjusted p-value < 0.05 and log_2_fold change >1 were considered DEGs. To generate volcano plots, the adjusted p-values were ranked from smallest to largest. For data analysis, ggplot and dplyr packages were used where the x-axis = log_2_(fold change) and the y-axis = -log_10_(adjusted p-value). Venn diagrams were generated using GraphPad Prism v.10.0.0.

### GO analysis and transcriptional pathways analysis

For functional analysis eggNOG-mapper [25] was used for KEGG and Gene ontology (GO) annotation, pfam_scan.pl [26] was used to predict the Pfam conserved domain. GO-enrichment analysis was performed using TBtools-II [27] in R (v.4.5.2) and R Studio software (v.2025.09.2 + 401) followed by using R studio package ggplot to identify the top 15 most significant gene functions for each data set. To identify the top 15 most significant gene functions for each data set, gene ratios were calculated as a ratio of gene counts in the selected set (adjusted p-value) and gene counts in the background (number of significant genes with the same function). For all data processed, adjusted p-values were labeled at a significant threshold of *p < 0.05* and log_2_(fold change) > 1.

### Statistical analysis

For all infection assays, GraphPad Prism (https://www.graphpad.com/) was utilized for statistical analysis. Outliers were removed using GraphPad Prism ROUT method with Q = 10%, cleaned data was used and two tailed unpaired student’s t-test with a 95% confidence interval, assuming Gaussian distribution.

### Data availability

The transcriptomic datasets generated in this study have been deposited in the NCBI database under BioSample accession number SAMN55364028 (https://www.ncbi.nlm.nih.gov/biosample/55364028.

## Results

### VW5 shows reduced reproduction compared to VW4 on susceptible tomato

**Fig 1:**
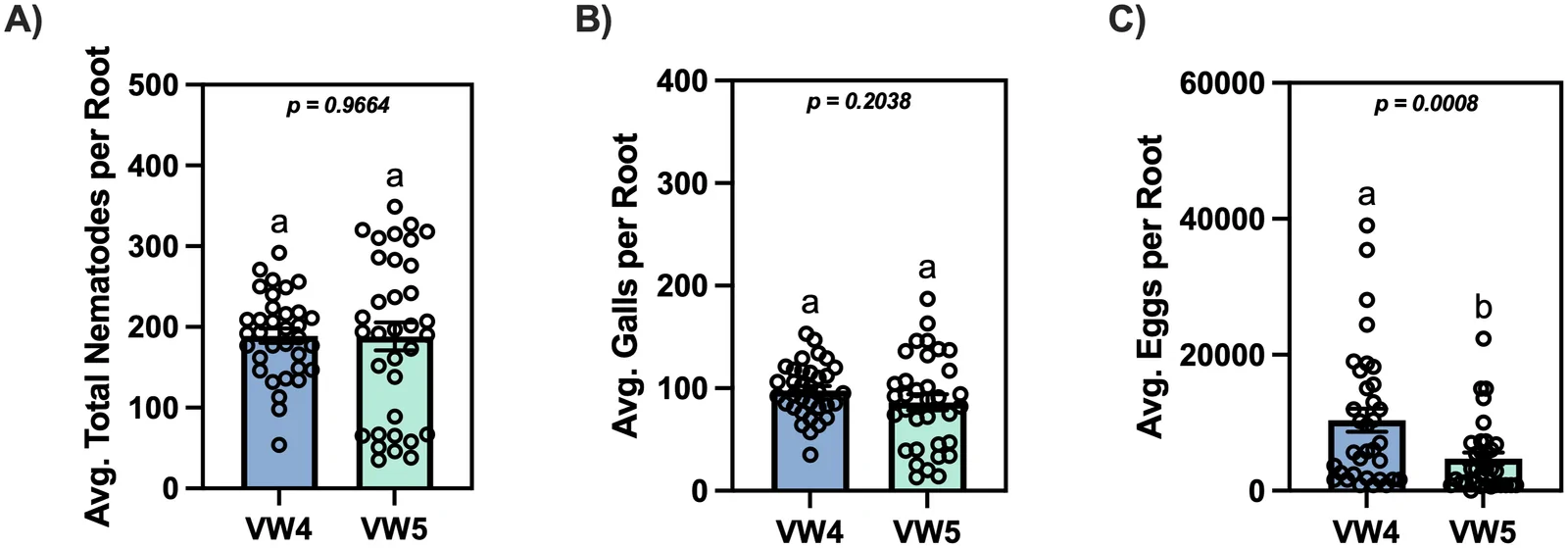
VW5 shows reduced reproduction compared with VW4 on susceptible tomato. Infection assays were performed on susceptible tomato cv. Moneymaker using *M. javanica* VW4 (wildtype, Mi-1–avirulent) and VW5 (Mi-1–virulent, resistance-breaking). Roots were harvested at 35 dpi.The following metrics were evaluated: **(A)** Average number of total nematodes (all developmental stages) per root system, **(B)** Average number of galls per root system, and **(C)** Average number of eggs per root system. Each data point represents an individual root system, and bars indicate means ± standard error of the mean (SEM). Data is pooled from three independent biological experiments (n = 35 roots per treatment). Statistical significance was determined using unpaired two-tailed Student’s t-tests (*p* < 0.05). Different letters indicate statistically significant differences between VW4 and VW5.

To evaluate whether *Mi-1*-virulence is associated with a fitness cost on susceptible hosts, we compared parasitism parameters of *M. javanica* VW4 and VW5 on susceptible tomato (cv. Moneymaker). Plants were harvested at 35 dpi for the average numbers of galls, nematodes (all life stages), and eggs per root, and fresh root weight were determined. Although several parameters showed differences among independent trials, only lower egg production in VW5 compared to VW4 was consistent across all assays (Fig 1A–1C and S1 Table). No significant difference in root weight was observed between genotypes (S2A Fig). Overall, this data identified a reduction in egg production as the most consistent and robust phenotype distinguishing VW5 from VW4 on susceptible tomato.

### VW5 exhibits reduced egg production compared to VW4 on cucumber and rice

To determine whether the reduced reproduction of VW5 occurred in other hosts besides tomato, we tested the VW4 and VW5 strains on additional susceptible hosts: cucumber (cv. Marketmore76) and rice (cv. Kitaake). Cucumber was selected because cucurbits are commonly used as rotation crops with tomato, whereas rice was included as a monocot host. Our analysis in tomato show that egg numbers were the most consistent and reproducible parameter distinguishing VW4 from VW5 (Fig 1A-1C and S1 Table). We therefore used egg production as the primary measure of reproductive fitness for cucumber and rice. Plants were harvested at 35 dpi, and fresh root weight along with nematode egg counts were quantified.

**Fig 2:**
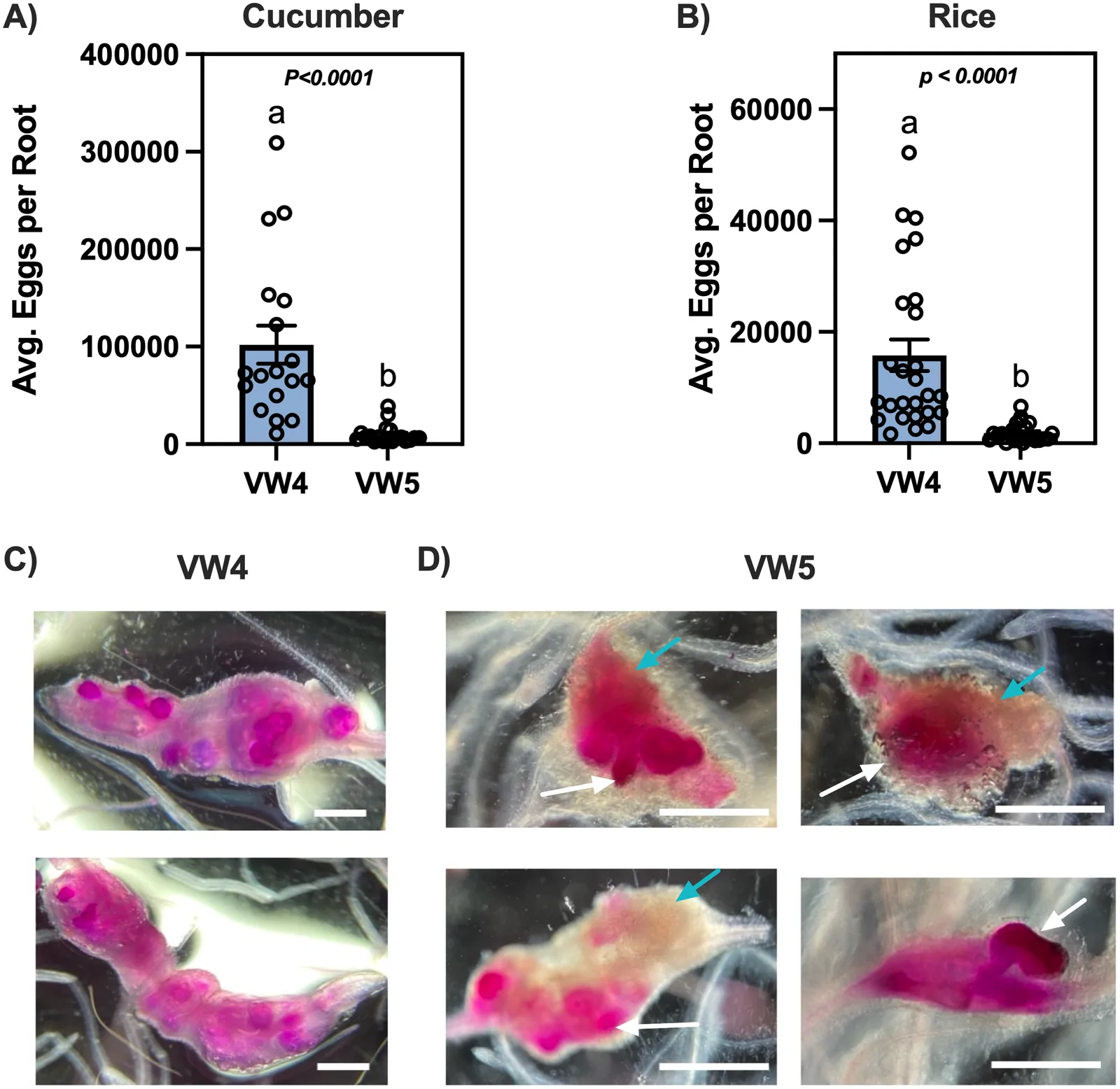
VW5 shows reduced egg production on cucumber and rice and induces abnormal gall morphology on cucumber. Infection assays harvested at 35 dpi evaluating the average egg numbers per cucumber root; **(A)** Average egg numbers per rice root **(B)**. Each data point represents an individual root system. Bars indicate means ± standard error of the mean (SEM). Statistical significance was determined using unpaired Student’s t-tests (*p* < 0.05), with p-values shown; different letters indicate statistically significant differences. Data are pooled from three independent biological experiments (cucumber, n = 18; rice, n = 26). Cucumber roots stained with Acid Fuchsin to visualize nematodes and gall morphology. Representative images show galls induced by VW4 (C) and VW5 **(D)**. VW5 female nematodes are indicated with white arrows, and dense cellular tissue is indicated in blue arrow. Scale bars = 350 µm.

As observed on tomato, VW5 produced significantly fewer eggs than VW4 on both cucumber and rice. Notably, the reduction in egg production was more pronounced with a 90% reduction in cucumber and an 88% reduction in rice than that found for susceptible tomato (58% reduction) (Fig 1C, 2A and 2B). Fresh root weight was recorded for tomato, cucumber and rice, and we found that there was a small, but significant difference for cucumber root weight between VW4 and VW5 infection but no difference for the other hosts (S2B and 2C Fig).

To assess gall morphology, we stained a subset of cucumber roots with Acid Fuchsin for nematode visualization. VW5-infected cucumber roots displayed pronounced abnormalities in gall morphology compared with VW4 (Fig 2C and 2D). At 35 dpi, galls produced by VW4 contained globular females embedded within the galled tissue with nematode heads aligned along vascular tissue (Fig 2C). Intriguingly, VW5 females were frequently found at the periphery of the gall, singular and often distorted, and galls contained dense callus-like tissue in regions where no females were present (Fig 2D). Rice roots were also stained; however, difficulty visualizing the nematodes within the roots precluded further morphological analysis.

### Attraction of VW4 and VW5 to host roots is not significantly different

**Fig 3:**
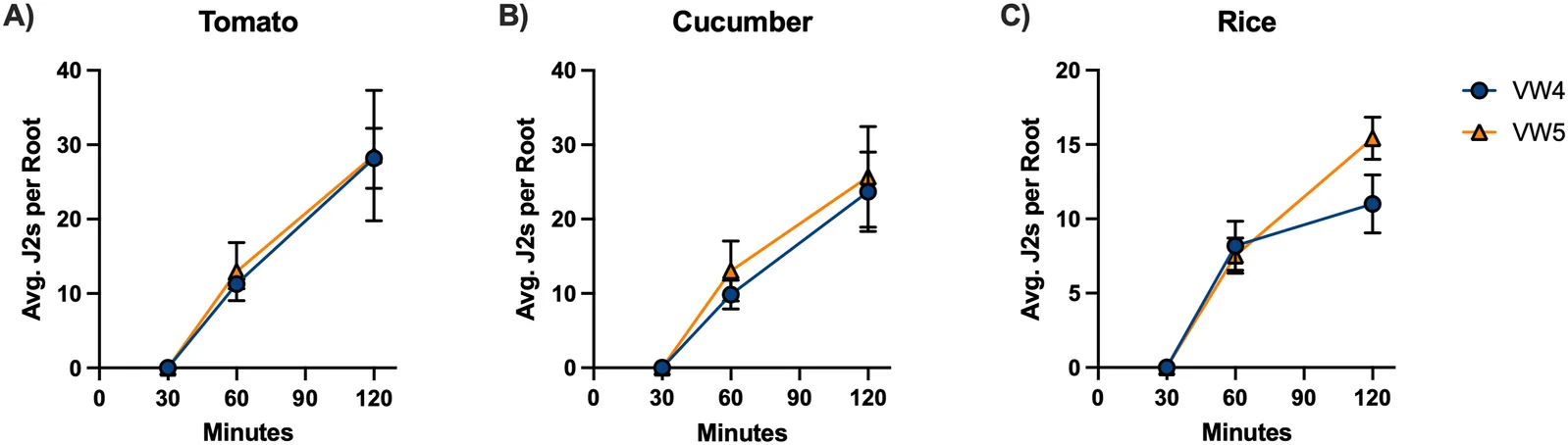
VW4 and VW5 show similar levels of root attraction on susceptible tomato, cucumber and rice. Root attraction assays counting the average number of J2s touching roots of tomato cv. Moneymaker **(A)**, cucumber cv. Marketmore76 **(B)**, and rice cv. Kitaake (C) after exposure for 30 minutes, 1 hour and 2 hours. Line graphs display J2s for VW4 (blue) and VW5 (yellow) with markers indicating means ± standard error of the mean (SEM, n = 3).

To assess whether impaired host finding of VW5 compared to VW4 contributed to the reduced reproductive success, we compared the attraction of pre-parasitic VW4 and VW5 J2s to tomato, cucumber, and rice roots using a Pluronic gel assay. VW4 and VW5 showed no significant differences between the two nematode strains for any host (Fig 3A–3C). These results indicate that VW5 retains the ability to find and move toward host roots and thus its reduced reproductive fitness is unlikely to result from a general defect in host finding.

### VW5 shows impaired feeding site development

To further investigate differences in gall morphology between VW4 and VW5, we examined transverse sections of galls and uninoculated roots by light and transmission electron microscopy. Galls were collected at 7 and 28 dpi from resistant tomato (cv. Motelle), susceptible tomato (cv. Moneymaker), and cucumber (cv. Marketmore76) infected with VW4 or VW5. Uninoculated tomato roots displayed typical root anatomy, including a single rhizodermis (epidermis) cell layer, a cortex composed of several cell layers, an endodermis, and a diarchal vascular cylinder with two primary xylem and two primary phloem bundles surrounded by a single layer of pericyclic cells (S3A and 3B Fig). Similarly, uninoculated cucumber roots showed parallel organization with only slight differences in endodermal cell morphology and a triarchic vascular cylinder with a central metaxylem vessel (S3C Fig). Above the root-hairs zone, the formation of secondary thickening commenced (S3D–3F Fig). The rhizodermis deteriorated and was replaced by the exodermis, and secondary phloem and xylem elements started to differentiate between primary xylem and phloem bundles. No formation of secondary cover tissue (periderm) was observed.

**Fig 4:**
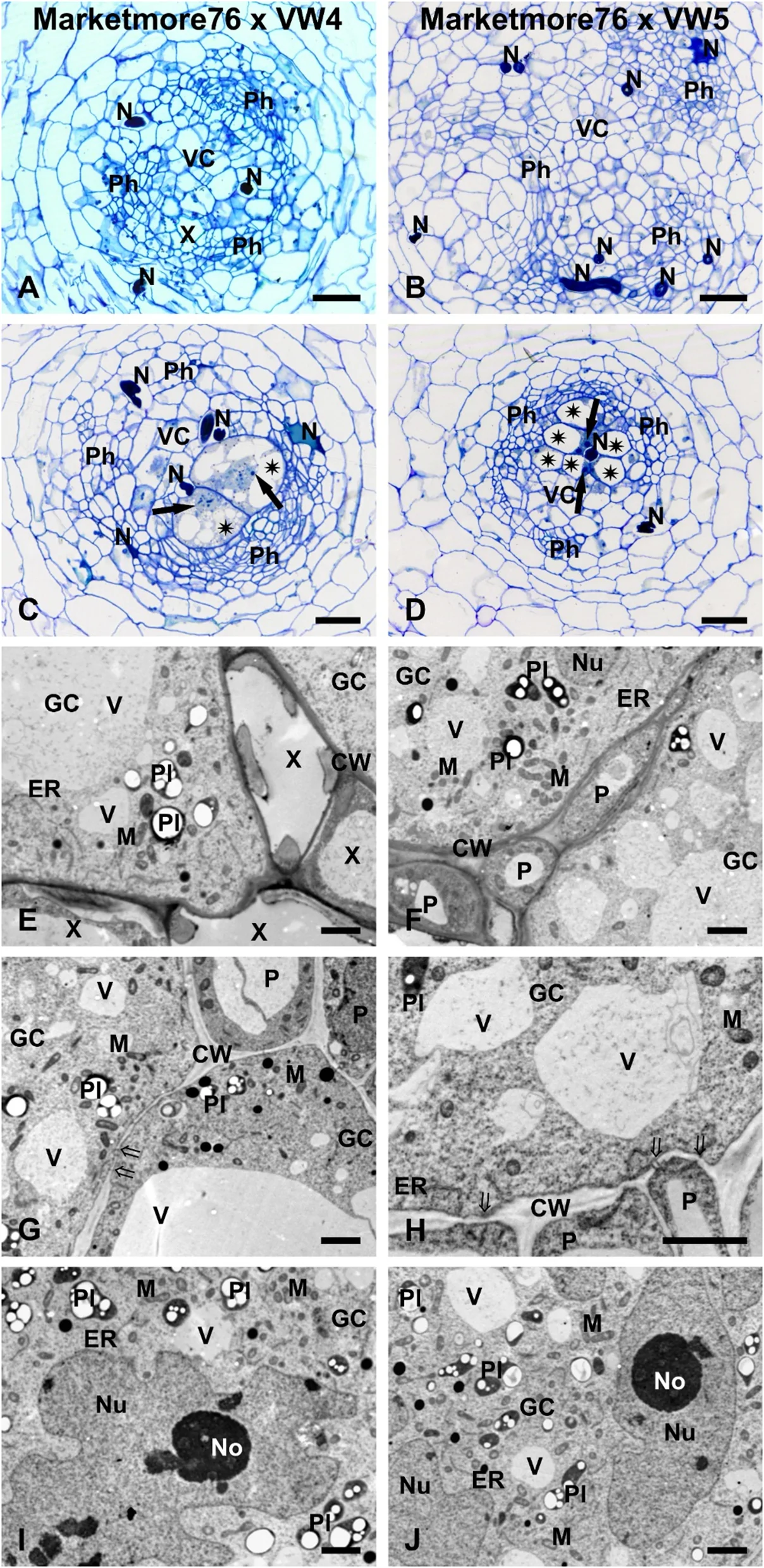
Anatomy and ultrastructure of giant cells developed in cucumber roots at 7 dpi. Light (A-D) and transmission electron microscopy (E-J) images of transverse sections of roots infected with VW4 strain (A, C, E, G and I) or VW5 strain (B, D, F, H and **J)**. Asterisks indicate giant cells **(C and D)**. Arrows point to giant cell nuclei **(C and D)**. Double tails arrows indicate plasmodesmata in thin fragments of giant cell walls **(G and H)**. Abbreviations: CW, cell wall; ER, endoplasmic reticulum; GC, giant cell; M, mitochondrion; N, nematode; No, nucleolus; Nu, nucleus; P, parenchymatic cell; Ph, phloem; Pl, plastid; X, xylem vessel; V, vacuole; VC, vascular cylinder. Scale bars: 50 µm (A-D) and 2 µm **(E-J)**.

Cross sections of galled cucumber roots were taken at the nematode head region and were only selected for comparisons of feeding site anatomy. For both nematode strains at 7 dpi, many invaded J2s had not established feeding sites in cucumber roots (Fig 4A and 4B). Presumably, the invasion took place in the root-tip regions and induced extensive cell divisions within the vascular cylinder without differentiation into GCs. However, some successful VW4 juveniles established groups of 2–3 clearly recognizable GCs (Fig 4C) whereas most VW5 juveniles were found surrounded by a ring of only enlarged cells still containing central vacuoles (Fig 4D).

**Fig 5:**
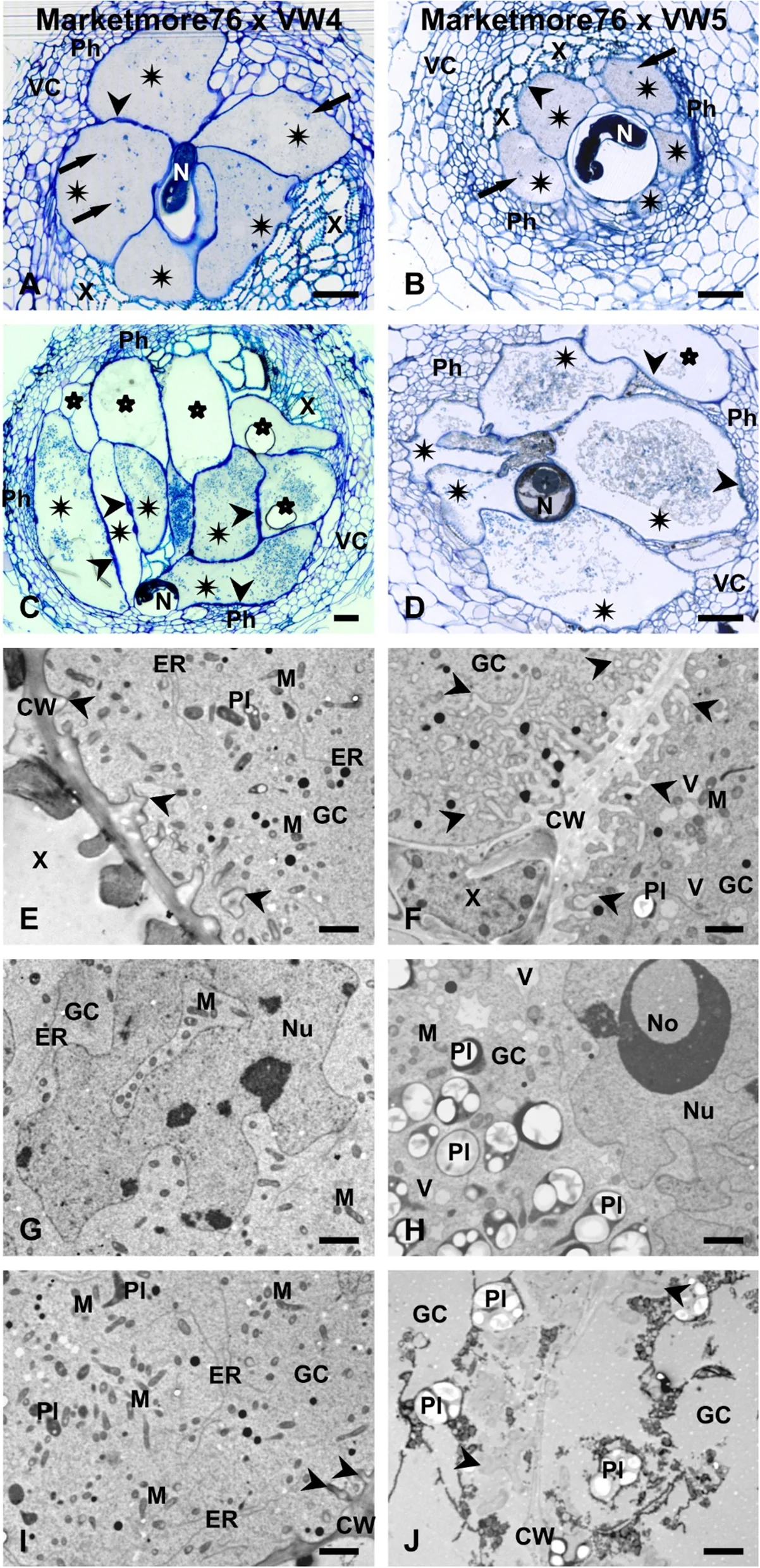
Anatomy and ultrastructure of giant cells developed in cucumber roots at 28 dpi. Light (A-D) and transmission electron microscopy (E-J) images of transverse sections of roots infected with VW4 strain (A, C, E, G and I) or VW5 strain (B, D, F, H, and **J)**. Asterisks indicate GCs in direct contact with juvenile (A-D) and stars indicate GCs without direct contact to nematode **(C-D)**. Arrows point go giant cell nuclei. Arrowheads indicate systems of cell wall ingrowths (A-F, I and **J)**. Abbreviations: CW, cell wall; ER, endoplasmic reticulum; GC, giant cell; M, mitochondrion; N, nematode; No, nucleolus; Nu, nucleus; Ph, phloem; Pl, plastid; X, xylem vessel; V, vacuole; VC, vascular cylinder. Scale bars: 50 µm (A-D) and 2 µm **(E-J)**.

In the case of VW5, the GCs or “enlarged cells” were usually surrounded by parenchymatic cells (Fig 4F and 4H) and only few xylem vessels contained protoplasm (Fig 5F). In contrast, VW4-induced GCs were frequently surrounded by fully differentiated xylem vessels (Fig 4E and Fig 5E), and no cell wall ingrowths were observed at 7 dpi but were present at 28 dpi GCs (Fig 4E–4H, and Fig 5E, 5F, 5I, and 5J). Some parts of the cell walls between the GCs and the neighboring parenchymatic cells remained thin and pierced with plasmodesmata at both time points (Fig 4G and 4H). Protoplasts of GCs contained vacuoles of different sizes at 7 dpi (Fig 4C–4J), but only small ones were present in GCs with non-degraded protoplasts at 28 dpi (Fig 5E–5H). The cytoplasm contained numerous mitochondria, plastids, and relatively few elements of the endoplasmic reticulum (Fig 4E–4J and Fig 5E–5l). Plastids and mitochondria had typical round or rod-shaped outlines, and no morphologically modified organelles were observed in cucumber but often occurred in GCs induced in tomato roots (S5D, S5G and S5I Fig).

Starch grains were abundant in plastids at 7 dpi (Fig 4E–4J). At 28 dpi, however, they were present in high numbers only in VW5-induced GCs (Fig 5E–5J). Starch is known to be a crucial nutrient source for nematode feeding, and saccharides are extensively mobilized to maintain an adequate nutritional supply [28]; [29]. Therefore, the persistence of abundant starch grains in VW5-induced GCs at 28 dpi suggests that nutrient acquisition by VW5 may be compromised during the later stages of feeding site development.

In general, the GC nuclei were strongly hypertrophied and contained electron-dense nucleoplasm and enlarged nucleoli for both time points and nematode strains (Fig 4I and 4J, and Fig 5G and 5H). Additionally, GCs nuclei became strongly amoeboid in outlines except for nuclei of GCs induced by VW5 at 7 dpi, which were hypertrophied but remained relatively round (Fig 4F and 4J).

At 28 dpi, two different patterns of feeding site anatomy could be discriminated between the two strains (**Fig 5A-5D**). Feeding sites were usually composed of 4–6 strongly hypertrophied GCs surrounding the nematode head and with extensive interfaces to the secondary xylem vessels (Fig 5A and 5B). The cell walls of the GCs were slightly thickened, and systems of cell wall ingrowths were weakly developed (Fig 5A, 5B, 5E, and 5F). If deposited, the cell wall ingrowths were usually formed at walls facing xylem vessels (Fig 5E, 5F, and 5I). GCs cytoplasm contained only small vacuoles, numerous mitochondria and plastids of typical round or elongated outlines (**Fig 5E-5I**). However, in some cases, feeding sites induced by both nematode strains contained additional GCs that did not contact the nematode head (Fig 5C and 5D). Such GCs contained usually poorly stainable and granular protoplast being degrading in the case of VW4 (Fig 5C and 5I) or degraded in the case of VW5 (Fig 5D and 5J).

**Fig 6:**
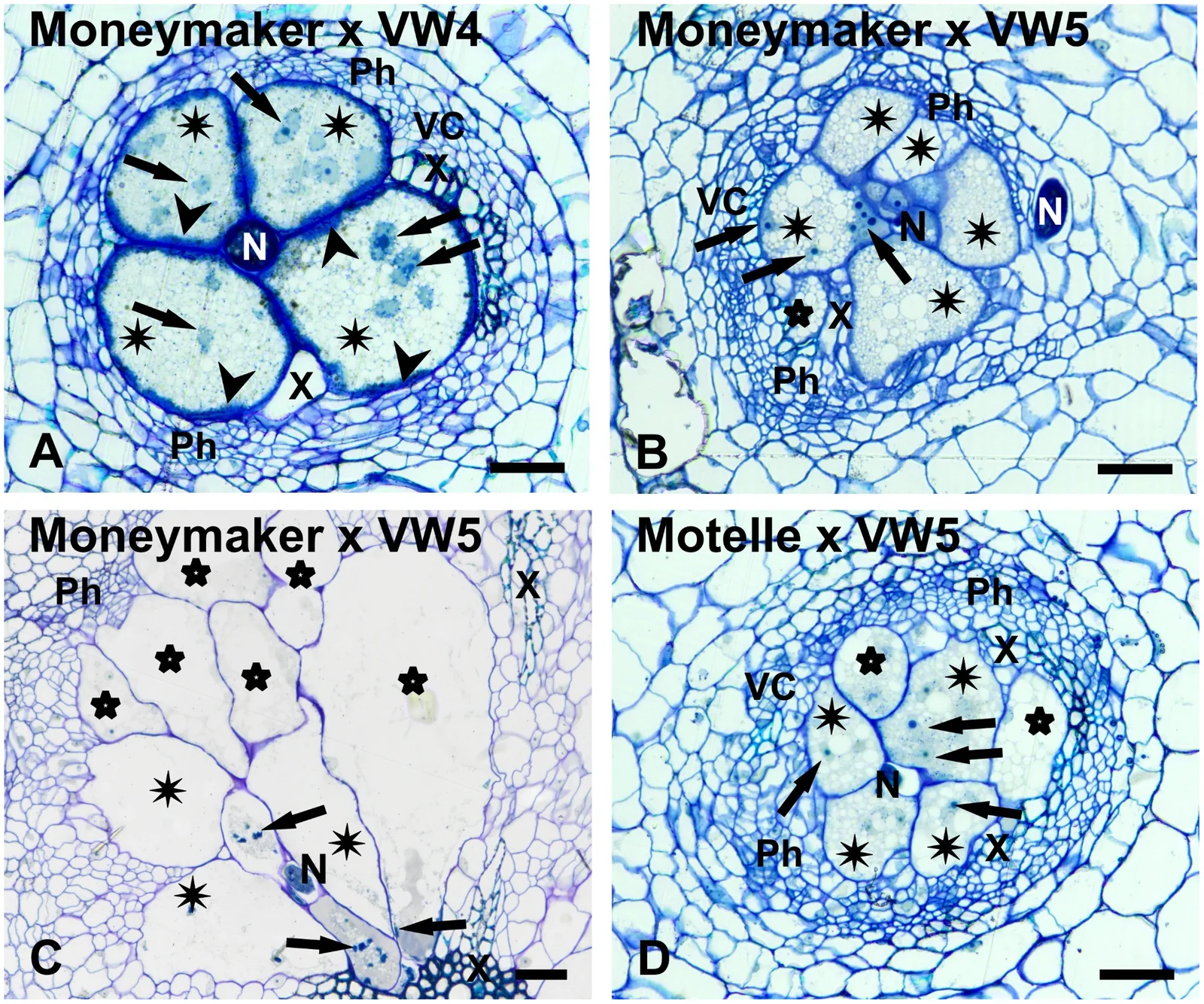
Anatomy of giant cells developed in tomato roots at 7 dpi. Light microscopy images of transverse sections of susceptible cv. Moneymaker (A-C) and resistant cv. Motelle (D) infected with avirulent VW4 strain (A) or virulent VW5 strain **(B-D)**. Asterisks indicate GCs in direct contact with juvenile (A-D) and stars indicate GCs without direct contact to nematode **(B-D)**. Arrows point to giant cell nuclei (A-D) and arrowheads indicate systems of cell wall ingrowths **(A)**. Abbreviations: N, nematode; Ph, phloem; X, xylem vessel; and VC, vascular cylinder. Scale bars: 50 µm.

**Fig 7:**
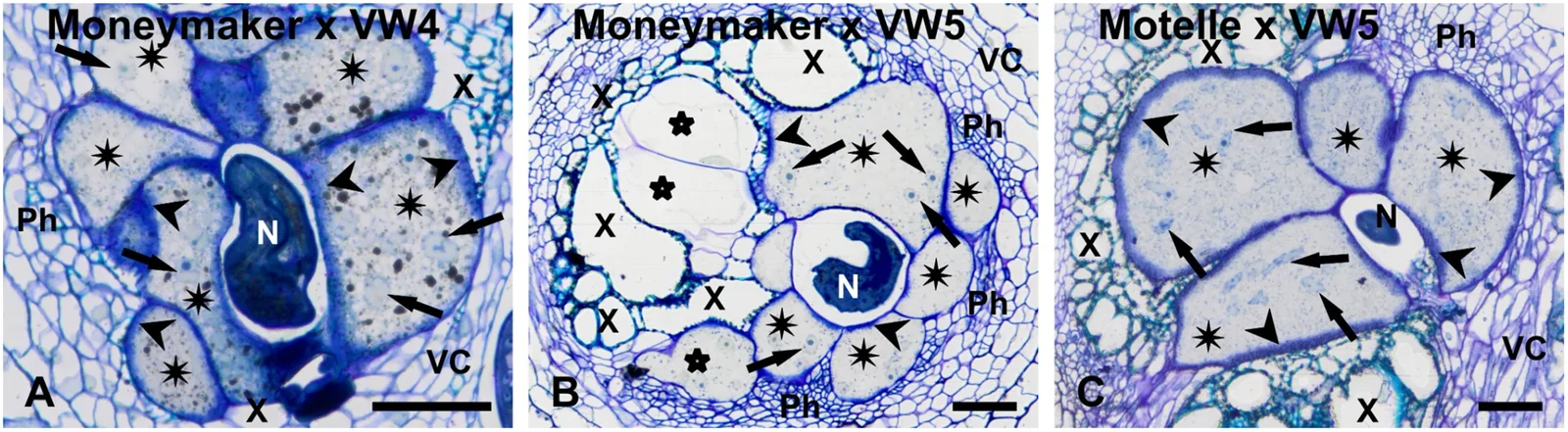
Anatomy of giant cells developed in tomato roots at 28 dpi. Light microscopy images of transverse sections of susceptible cv. Moneymaker (A and B) and resistant cv. Motelle (C) infected with avirulent VW4 strain (A) or virulent VW5 strain **(B and C)**. Asterisks indicate GCs in direct contact with juvenile (A-C) and stars indicate GCs without direct contact to nematode **(B)**. Arrows point to giant cell nuclei and arrowheads indicate systems of cell wall ingrowths. Scale bars: 50 µm. Abbreviations: N, nematode; Ph, phloem; X, xylem vessel; VC, vascular cylinder. Scale bars: 50 µm.

VW4 and VW5 feeding sites in tomato displayed similar results, however at 7 dpi feeding site initiation and development was more progressed than observed in cucumber galls for both nematode strains. Cross sections revealed that GCs were consistently located within the vascular cylinder adjacent to conductive elements of xylem and phloem (Fig 6A–6D and 7A–7C). Infected roots showed marked proliferation of vascular cylinder cells relative to uninfected roots (Fig 6A–6D vs S3A and S3B Fig). During VW5 parasitism of susceptible tomato, GCs were frequently displaced toward the periphery of expanded vascular cylinders, with limited contact surface to the conductive elements (Fig 6C).

Typical feeding sites induced in Moneymaker roots by VW4 juveniles contained 4–6 strongly hypertrophied GCs surrounding the nematode head (Fig 6A) and the number of GCs rose to 6–8 at 28 dpi (Fig 7A). In contrast, VW5 infections on both Moneymaker and Motelle frequently contained more than 6 GCs, including additional GCs not in direct contact with the nematode body, giving the impression of a second GCs ring (Fig 6B–6D). Despite higher GC numbers, VW5-induced GCs were usually less hypertrophied and rather thin-walled than those induced by VW4 at both 7 and 28 dpi (Fig 6A–6D, and Fig 7A–7C).

VW4-induced GCs exhibited extensive cell wall thickening and branched cell wall ingrowths between GCs and adjacent xylem vessels and sieve tubes (Fig 6A, and S4A, S4D and S4G Fig), which became more pronounced at 28 dpi (Fig 7A, and S5A and 5D Fig). In contrast, cell wall ingrowths were reduced or absent in VW5-induced GCs, particularly in susceptible Moneymaker roots (Fig 6B and 6C, and S4B and S4C Fig; Fig 7B and 7C, and S5B and S5C Fig). Thin regions of cell wall containing plasmodesmata were frequently observed between GCs in VW4 and VW5 infections at both time points (S4D, S4E and S4I Fig, and S5D and S5F Fig).

At the ultrastructural level, cytoplasm of VW4-induced GCs was uniformly electron dense at both 7 and 28 dpi. It was rich in organelles, including mitochondria, plastids, limited endoplasmic reticulum (ER) structures, hypertrophied nuclei, and numerous small vacuoles which were evenly distributed inside the GCs (S4A, S4G and S4J Fig and S5G and S5J Fig). Most of the mitochondria were not morphologically changed, however, at 28 dpi many of them acquired elongated, circular or cup-like shapes (S4A and S4G Fig, and S5D, S5G and S5J Fig). Plastids appeared numerously and rarely contained small starch grains (S4A, S4D, S4G and S4J Fig, and S5D, S5G and S5J Fig). ER cisternae were rare and usually formed short structures at both time points (S4A, S4D and S4G Fig, and S5A and S5D Fig). GC nuclei were strongly hypertrophied, amoeboid in outlines, and contained uniformly electron dense nucleoplasm with prominent nucleoli at 7 and 28 dpi (S4G and S4J Fig, and S5J Fig).

In contrast, the cytoplasm of GCs induced by VW5 in Moneymaker was frequently electron-translucent and usually contained large vacuoles resembling the central vacuole (S4B, S4E, S4H and S4K Fig). Nevertheless, at 28 dpi these vacuoles were reduced in size, and two zones of cytoplasm could be discriminated: the opaquer layer next to the cell walls which contained the bulk of organelles and the transparent central part containing only vacuoles of different shapes and sizes (Fig 7B, and S5E and S5H **Fig**). The mitochondria and plastids were less numerous (S4B, S4E, S4H and S4K Fig, and S5B, S5E, S5H and S5K Fig) and were not changed morphologically at any time point. Additionally, plastids usually contained small starch grains (S4B and S5B Fig). ER cisternae were more numerous than observed in VW4 and were often arranged in circular structures in 7 dpi and 28 dpi GCs (S4B and S4H Fig, and S5B and S5H Fig). In contrast, nuclei were slightly hypertrophied and appeared as round forms with small bulges at both time points, when compared to amoeboid in GCs induced by VW4 (S4H and S4J Fig, and S5E, S5J and S5K Fig).

VW5-induced GCs in resistant cv. Motelle showed resemblance as observed in VW4-induced GCs, especially at 28 dpi (Fig 6A and 6D, and S4A and S4C Fig; Fig 7A and 7C, and S5A and S5C Fig). The GCs cytoplasm displayed similar opacity and contained numerous small vacuoles (S4C, S4F and S4I Fig, and S5C, S5F, S5I and S5L Fig). In addition, they contained abundant mitochondria, which often acquired elongated or ring-like shapes at 28 dpi (S5C, S5 and S5I Fig). Many plastids contained large starch granules. Similarly, nuclei were strongly hypertrophied, amoeboid in shape, and contained prominent nucleoli at 7 and 28 dpi (S4L Fig and S5L Fig). Together, these light and transmission electron microscopy analyses demonstrate that VW5 establishes structurally compromised feeding sites during infection on susceptible hosts, characterized by reduced GCs hypertrophy, limited vascular connectivity, and altered organization of GCs protoplast, which likely leads to premature degradation of its feeding sites, restricts its nutrient supply and contributes to their reduced reproduction.

### Transcriptomes of cucumber galls reveal weaker host reprogramming by VW5 compared to VW4 at early infection

**Fig 8:**
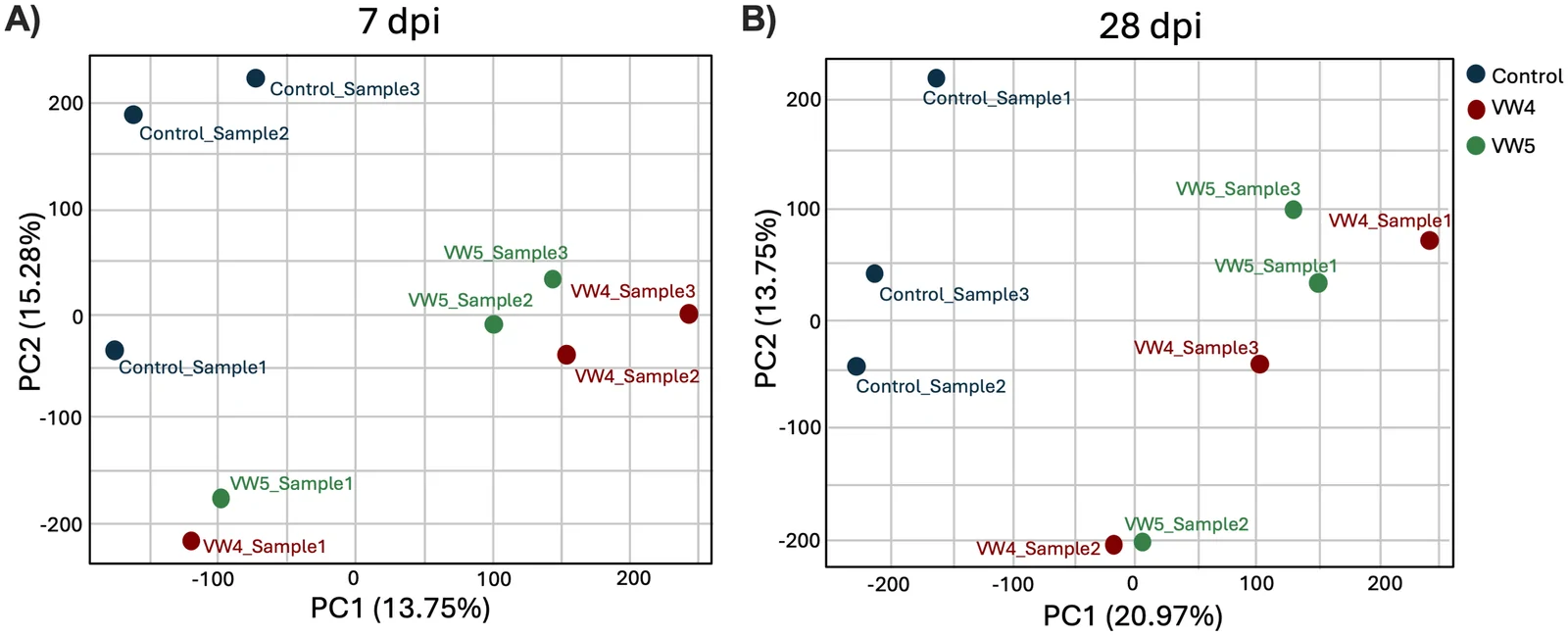
Principal component analysis distinguishes infected from uninfected cucumber roots at early and late infection stages. Principal component analysis (PCA) of mRNA-seq data shows clear separation between uninfected (control) cucumber roots and roots infected with *M. javanica* VW4 or VW5 at both 7 (A) and 28 dpi **(B)**. The x- and y-axes show Principal component 1 and Principal component 2, which explains 20.97% and 15.2% of the total variance, respectively. Each data point represents an independent biological replicate of RNA extracted from galled tissue of cucumber roots infected with VW4 or VW5, or from uninoculated control roots. Black data points indicate uninoculated control roots, red indicates VW4-infected roots, and green indicates VW5-infected roots.

Because VW5 showed reduced egg production across tomato, cucumber, and rice, we next sought to identify host processes associated with this fitness defect. Among the hosts examined, cucumber displayed the strongest and most consistent differences in gall and feeding-site morphology between VW4 and VW5, providing the clearest biological contrast for downstream molecular analysis. We therefore selected cucumbers to investigate transcriptional changes associated with defective feeding-site development and reduced nematode reproduction. RNA-seq was performed on cucumber galls induced by VW4 or VW5 at 7 and 28 days post-inoculation and compared with anatomically comparable root segments collected from uninoculated plants. Principal component analysis (PCA) showed partial separation between infected and uninoculated samples at both time points, consistent with infection-associated variation in host gene expression, although PC1 and PC2 explained only a modest proportion of the total variance (Fig 8A and 8B). In addition, VW4- and VW5-infected samples clustered closer to each other than to uninoculated controls, indicating a substantial shared host response to nematode infection together with strain-associated transcriptional differences.

**Fig 9:**
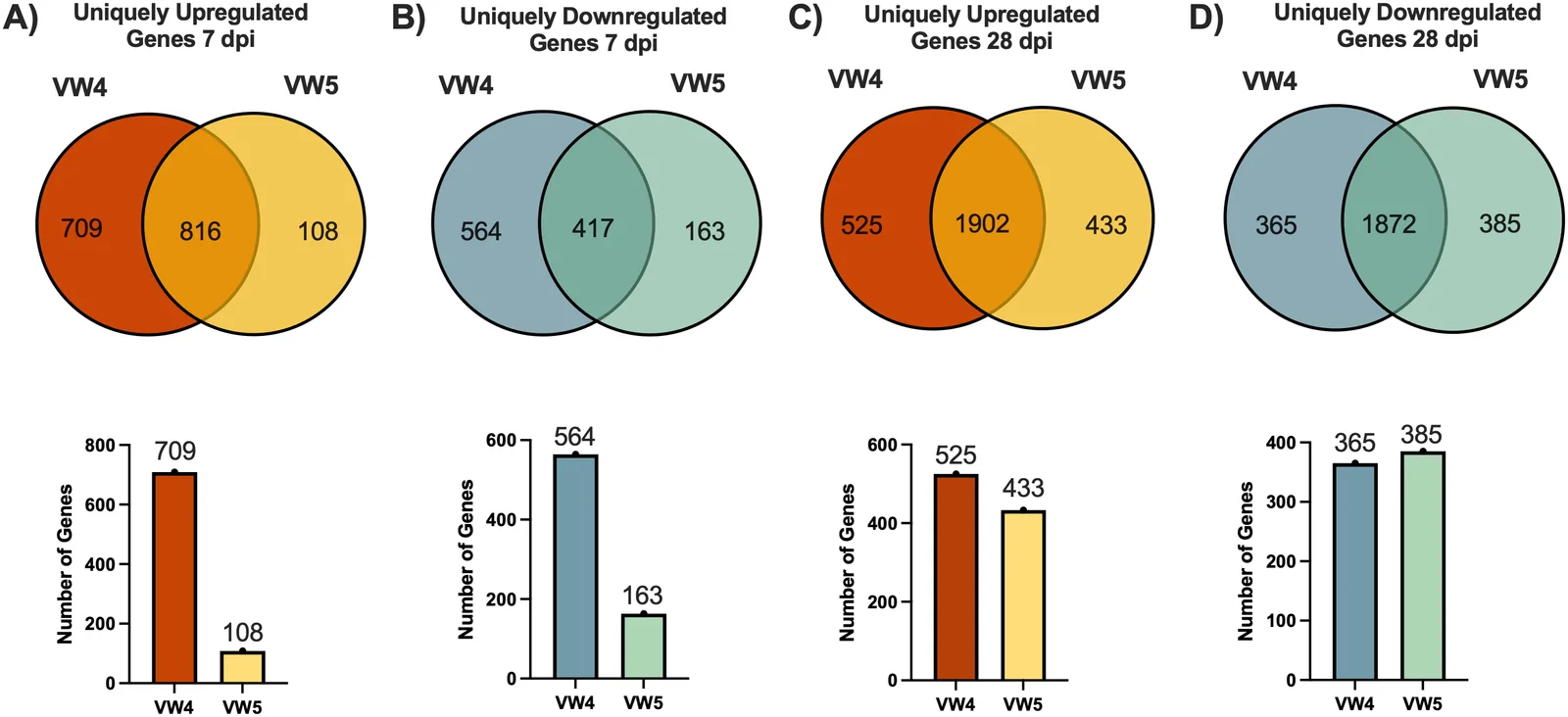
Differentially expressed cucumber genes unique to VW4 or VW5 show greater divergence during early infection. Venn diagrams showing significantly upregulated genes at 7 dpi **(A)**, significantly downregulated genes at 7 dpi **(B)**, significantly upregulated genes at 28 dpi **(C)**, and significantly downregulated genes at 28 dpi **(D)**. Numbers indicate genes uniquely regulated in either VW4 or VW5, while overlapping regions represent genes significantly regulated in both. Bar graphs summarizing the number of uniquely up- and downregulated genes in VW4 and VW5 at 7 and 28 dpi. Cool colors represent downregulated genes, and warm colors represent upregulated genes.

At 7 dpi, VW4 infection resulted in extensive transcriptional reprogramming, with 1,525 genes significantly upregulated and 981 genes downregulated relative to uninoculated controls (adjusted p-value < 0.05; log_2_(fold change) > 1); S1 and S2 Data, and S6 Fig). In contrast, VW5 infection at the same time point resulted in 924 upregulated and 580 downregulated genes, indicating an attenuated early host transcriptional response during VW5 infection (adjusted p-value < 0.05; log_2_(fold change) > 1); S3 and S4 Data, and S6 Fig). After excluding DEGs shared between the VW4- and VW5-versus-control comparisons, VW4 showed 709 uniquely upregulated and 564 uniquely downregulated genes, whereas VW5 showed only 108 uniquely upregulated and 163 uniquely downregulated genes (Fig 9A and 9B; S5–S6 and S9–S10 Data).

**Fig 10:**
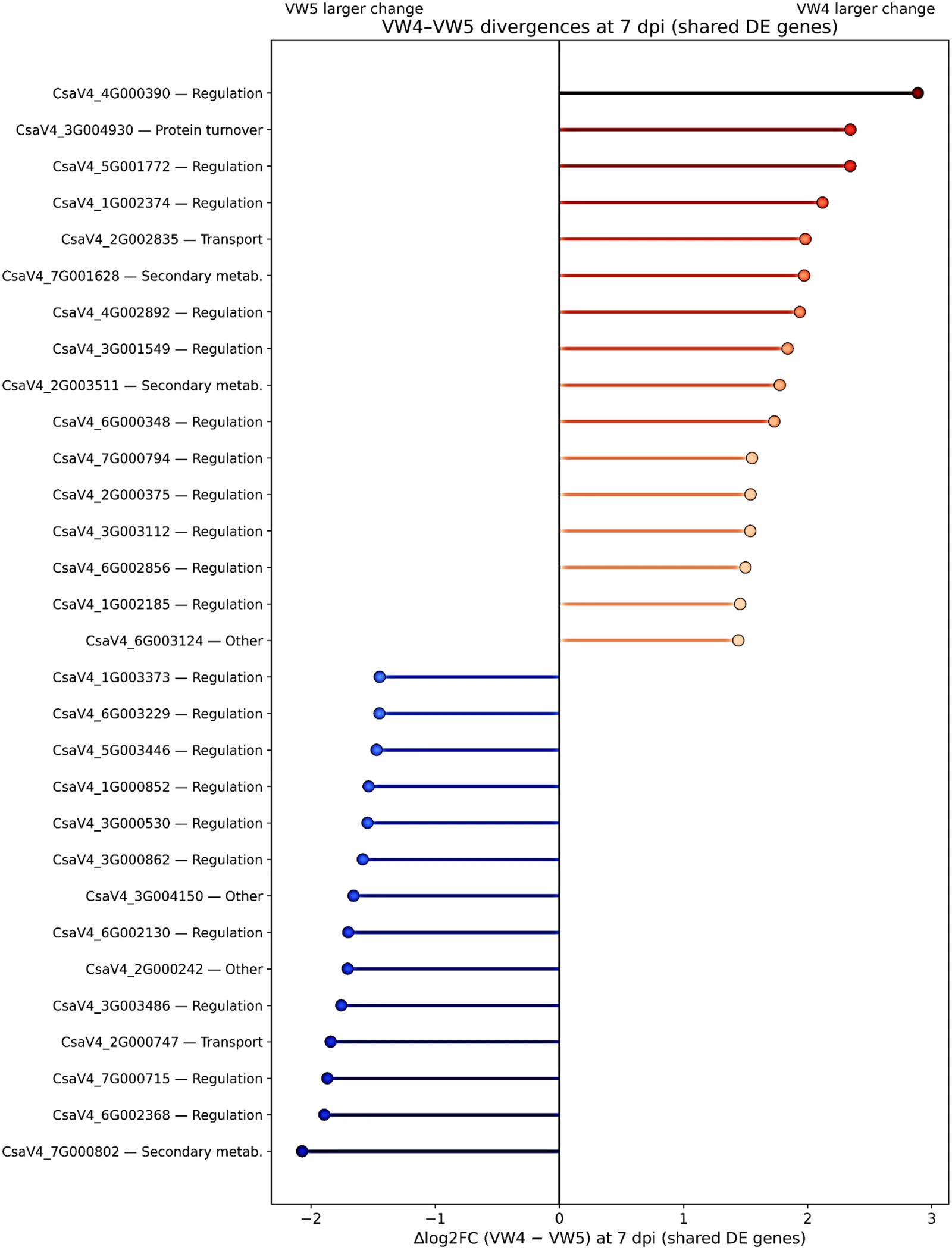
Divergence in host cucumber gene expression changes between VW4 and VW5 at 7 dpi. Lollipop plot shows the difference in log2 fold-change between strains for genes that were significantly differentially expressed in both VW4- and VW5-induced galls at 7 dpi (each compared to uninoculated controls). The x-axis shows Δlog2FC = log2FC(VW4) − log2FC(VW5); positive values indicate genes with larger expression changes in VW4, whereas negative values indicate larger expression changes in VW5. Each point represents one gene, and lines emphasize the magnitude of Δlog2FC. Labels show the gene ID and a brief functional category.

**Fig 11:**
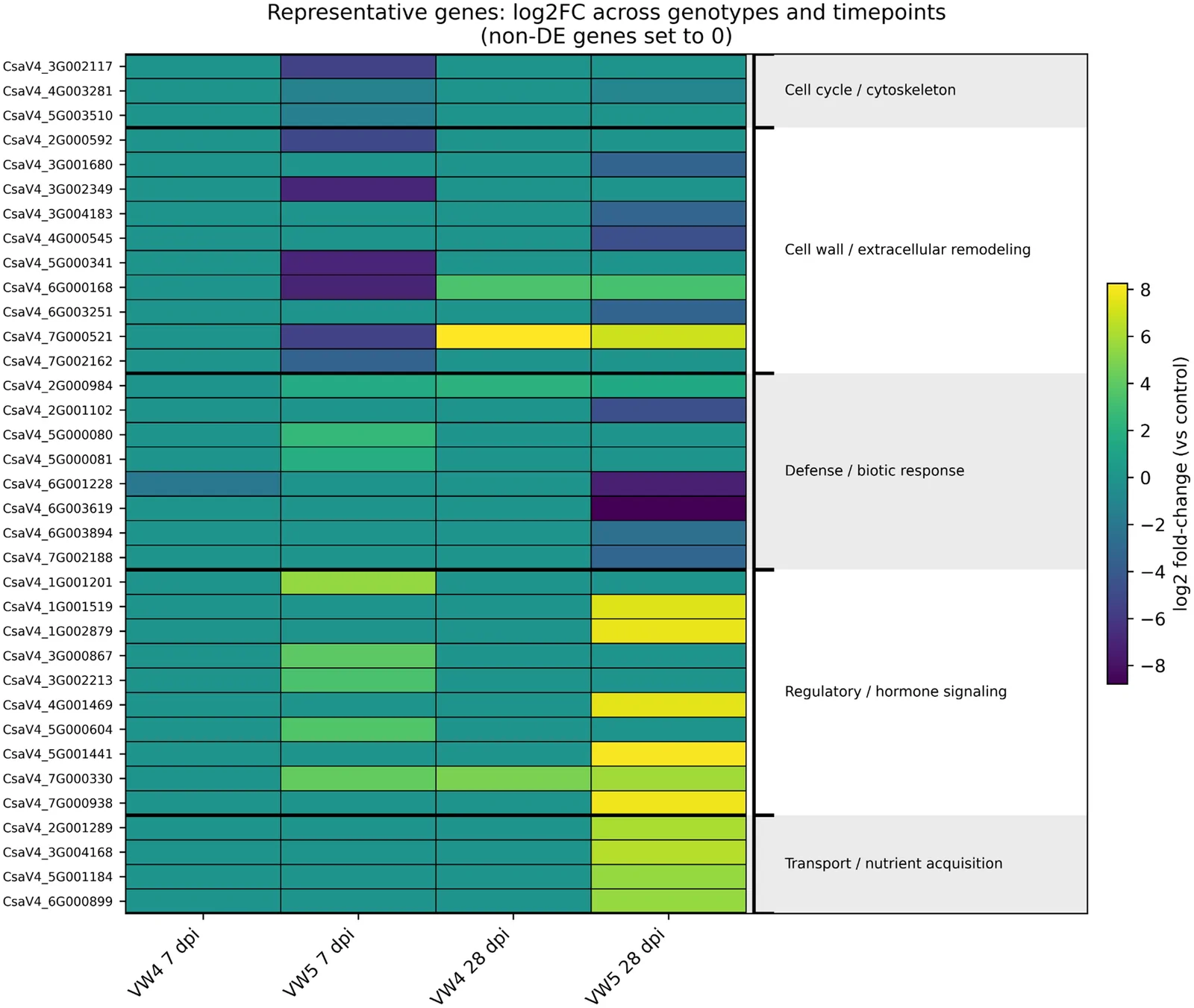
Representative gene expression changes in VW4- and VW5-induced cucumber galls at 7 and 28 dpi. Heatmap displays a continuous color gradient of log2 fold-change (vs uninoculated controls) for a representative set of genes selected from the VW5 strain-unique differential expression lists (7 dpi and/or 28 dpi). Genes are grouped into broad functional categories indicated on the right. For visualization, genes not significantly differentially expressed in each comparison are shown as 0.

To determine whether VW4 and VW5 differed only in the size of the response or other parameters including the magnitude of shared changes, we compared log2 fold-changes for overlapping differentially expressed genes (DEGs) in both infections at 7 dpi. Overall, shared DEGs showed slightly larger expression changes in VW4 than VW5, although this difference was not uniform across functional groups (Fig 10). Genes associated with cell wall/extracellular remodeling showed a clear bias toward larger changes in VW4 than VW5 at 7 dpi, whereas secondary metabolism genes, in aggregate, showed relatively larger changes in VW5 (Fig 10). Next, we evaluated excluded overlapping DEGs between the strains to identify unique DEGs at each time point. Notably, at 7 dpi, the VW5-uniquely downregulated gene set included transcripts associated with transport/nutrient acquisition, regulatory and hormone-associated pathways, as well as defense/biotic response and secondary metabolism. However, the most striking feature was strong repression of host genes involved in cell-wall and extracellular remodeling, processes required for feeding-site establishment and gall expansion. Among the most strongly regulated, VW5-uniquely downregulated transcripts were Expansin-A7 (*CsaV4_5G000341*), Callose synthase 5 (*CsaV4_3G002349*), Xyloglucan endotransglucosylase/hydrolase XTH25 (*CsaV4_2G000592*), Laccase-4/IRX12 (*CsaV4_6G000168*), and LRR–extensin LRX6 (*CsaV4_7G000521*). These genes encode cell wall-loosening enzymes, polysaccharide synthesis/remodeling factors, and structural wall components that collectively support expansion and function of hypertrophied giant cells (Fig 10 and 11; S5 and S6 Data).

**Fig 12:**
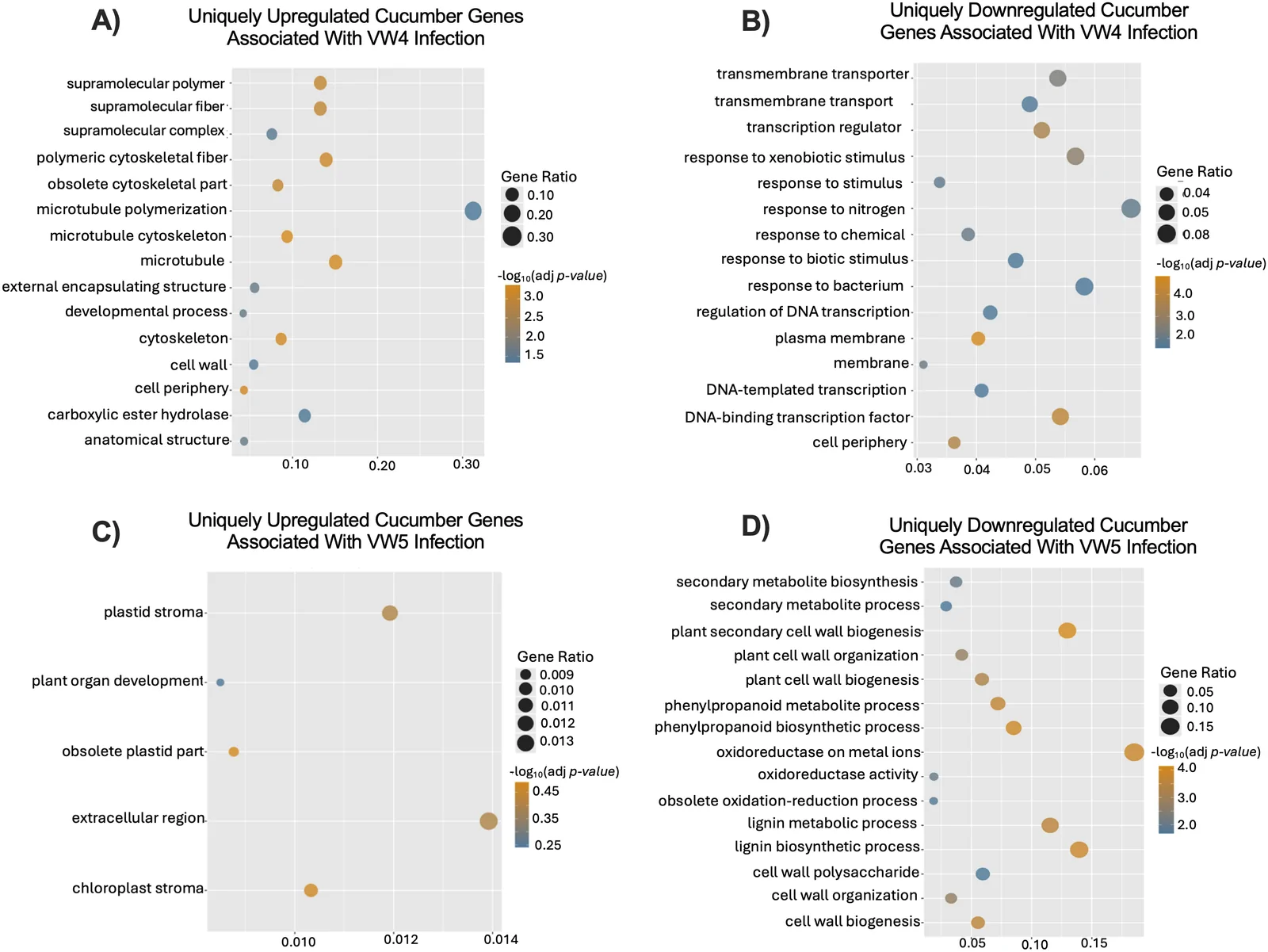
GO enrichment analysis of VW4 and VW5 uniquely differently expressed cucumber genes at 7 dpi. GO enrichment analysis was performed using genes uniquely up- or downregulated in cucumber roots infected with VW4 or VW5 strain of *M. javanica* at 7 dpi, relative to each other. Only genes that were significantly regulated respective to VW4 or VW5 were included in the analysis. VW4-uniquely upregulated genes **(A)**, VW4-uniquely downregulated genes **(B)**, VW5-uniquely upregulated genes **(C)**, and VW5-uniquely downregulated genes **(D)**. GO biological process terms are shown on the y-axis, and gene ratio is shown on the x-axis. Circle size represents the number of genes associated with each GO term (condensed to fit on chart), and circle color indicates significance based on −log_10_(adjusted p-value). The top 15 most significantly enriched GO terms are displayed.

GO enrichment analysis supported this interpretation at the pathway level. At 7 dpi, VW4-unique genes were enriched for GO terms representing biological processes required for feeding site formation, including cellular reorganization, vascular development, cell cycle related processes, and modulation of host defense-related pathways (Fig 12A and 12B, and S7A Fig) [7,30]. In contrast, VW5-unique genes at 7 dpi showed limited enrichment for feeding site–associated processes and were primarily enriched for secondary metabolic and plastid-related functions, indicating an impaired ability to reprogram host tissues morphogenesis during early infection (Fig 12C and 12D, and S7B Fig).

By 28 dpi, the overall magnitude of transcriptional reprogramming increased for both strains. VW4 infection resulted in 2,427 upregulated and 2,237 downregulated genes, while VW5 infection resulted in 2,335 upregulated and 2,257 downregulated genes (Fig 9C and 9D; S6 Fig). Analysis of uniquely regulated genes at this time point showed comparable numbers of strain-specific genes between VW4 and VW5, including 525 uniquely upregulated and 365 uniquely downregulated genes for VW4 and 433 uniquely upregulated and 385 uniquely downregulated genes for VW5 (Fig 9C and 9D; S7–S8 and S11–S12 Data). The substantially larger overlap between VW4 and VW5 DEGs at 28 dpi relative to 7 dpi is explained by the reduced number of VW4-specific DEGs at the later infection stage, rather than by a broad convergence of the VW4- and VW5-induced host responses(S7 Fig).

**Fig 13:**
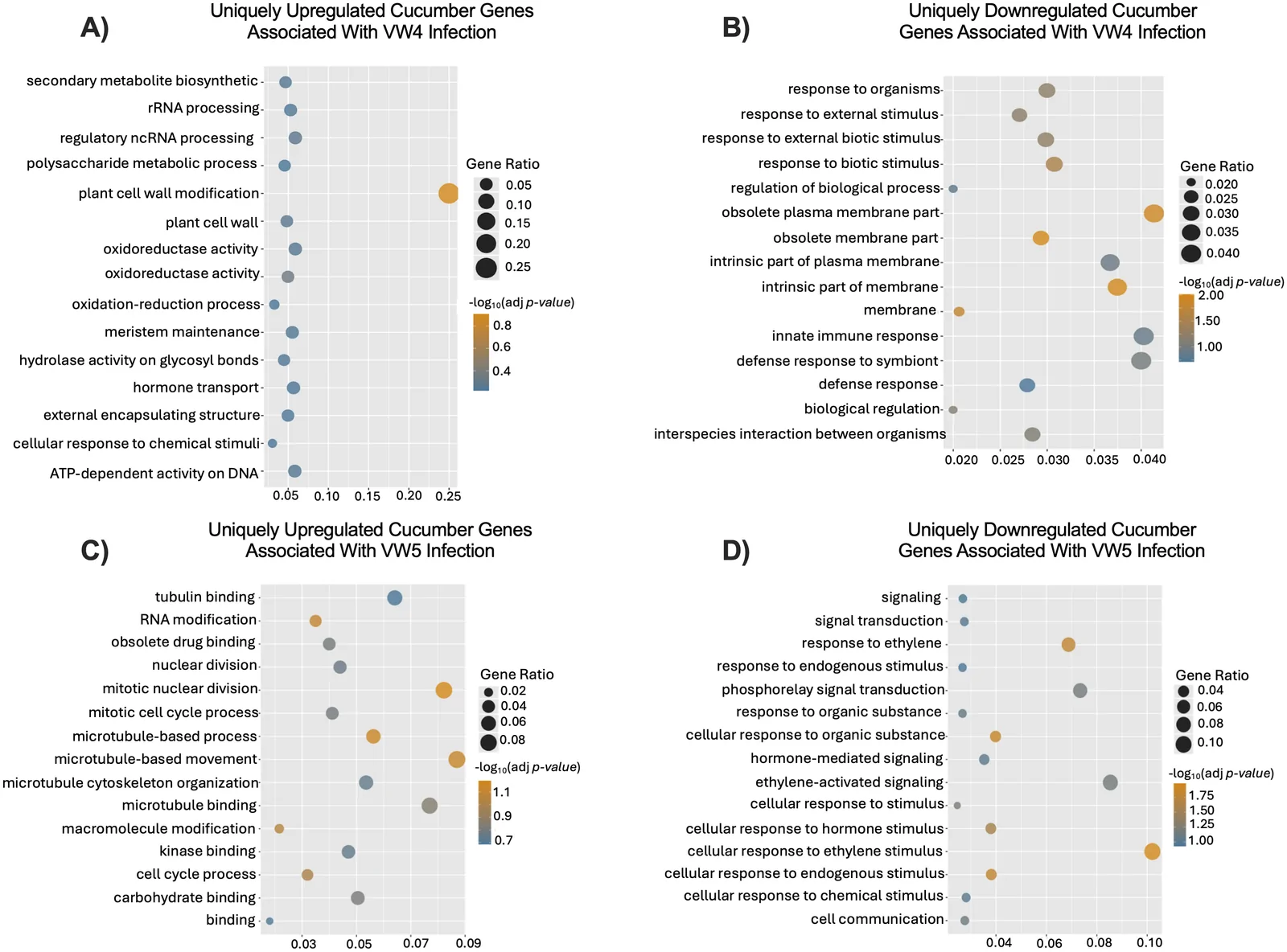
GO enrichment analysis of VW4-uniquely differentially expressed cucumber genes at 28 dpi. GO enrichment analysis was performed using genes uniquely up- or downregulated in cucumber roots infected with VW4 or VW5 strain of *M. javanica* at 28 dpi, relative to each other. Only genes that were significantly regulated respective to VW4 or VW5 were included in the analysis. VW4-uniquely upregulated genes **(A)**, VW4-uniquely downregulated genes **(B)**, VW5-uniquely upregulated genes **(C)**, and VW5-uniquely downregulated genes **(D)**. GO biological process terms are shown on the y-axis, and gene ratio is shown on the x-axis. Circle size represents the number of genes associated with each GO term (condensed to fit on chart), and circle color indicates significance based on −log_10_(adjusted p-value). The top 15 most significantly enriched GO terms are displayed.

GO enrichment at 28 dpi indicated that VW4-uniquely upregulated genes were enriched for processes associated with feeding site maintenance, cell wall regulation, and metabolic activity, including RNA processing, hormone transport, meristem maintenance, and ATP-dependent activities (Fig 13A; S7C Fig). In contrast, VW4-uniquely downregulated genes were enriched for defense- and stimulus-response biological processes, consistent with downregulation of immune-related pathways at this stage (Fig 13B; S7D Fig). VW5-uniquely upregulated genes at 28 dpi were also enriched for processes associated with feeding site function and hormone-response pathways; however, many of these processes overlapped with those enriched among VW4-unique genes at 7 dpi, supporting a temporal delay in activation of feeding site–associated pathways during VW5 infection (Fig 12A and 13C). As examples, the VW5-uniquely upregulated genes at 28 dpi included transcription factors (e.g., ERF086/CsaV4_3G001489 and NAC043/CsaV4_6G000905) and enzymes linked to lipid and related metabolic pathways (e.g., fatty acyl-CoA reductase/CsaV4_1G001519 and a BAHD acyltransferase/CsaV4_6G000771) (Fig 10 and 11). Notably, unlike VW4, VW5-uniquely downregulated genes at 28 dpi did not show enrichment for defense-related GO terms, suggesting limited strain-specific suppression of defense-associated processes at this stage (Fig 13B and 13D).

## Discussion

In this study, we demonstrate that Mi-1 resistance-breaking in *M. javanica* strain VW5 is associated with a measurable reduction in fitness on susceptible hosts compared to its avirulent progenitor VW4. Specifically, VW5 produced fewer eggs than VW4 on three susceptible hosts: tomato, cucumber, and rice. This reduction in reproduction was not attributable to impaired root attraction, as both strains attracted to host roots at comparable rates. However, nematode development was delayed in VW5 compared to VW4. Notably, the reduction in egg production was more pronounced in rice and cucumber than in tomato.

In both susceptible tomato and cucumber, microscopic examination revealed that VW5 is compromised in its ability to establish and maintain functional feeding sites. Differences in feeding site initiation and development were already discernable by 7 dai. At this stage, VW5-induced GCs were less developed in both hosts. Defects included reduced cellular hypertrophy, altered GC organization, limited vascular connectivity, and poorly developed cell wall ingrowths. The phenotype was particularly pronounced in cucumber, consistent with the greater reduction in egg production observed on this host. Because giant cells are essential for sustained nutrient acquisition, these structural defects likely underlie the reduced fecundity of VW5.

It is generally accepted that each GC arises from a single strongly hypertrophied and structurally modified cambial, meristematic, or parenchymatic cell and contains numerous enlarged and amoeboid nuclei generated through endopolyploidization and amitotic divisions [30–32]. The increase in nuclear numbers is most rapid during the first 7 days after inoculation, when the number of nuclei doubles each day, whereas no mitotic activity occurs in GCs associated with adult nematodes [33]. In this context, the relatively round outlines and low degree of hypertrophy of GC nuclei in feeding sites induced by VW5 in tomato and cucumber at 7 dpi (Fig 4 and **S4 Fig**), together with the enrichment of uniquely upregulated GO terms related to microtubule organization, mitosis, and cell cycle-associated processes in VW5 feeding sites, may indicate a relative delay in GC development. In other words, whereas VW4-induced GCs appear to function as fully established nutrient-providing structures, those induced by VW5 may still be in the process of establishment and differentiation.

Transcriptomic analyses further indicate that VW5 is impaired in its ability to reprogram host gene expression. At 7 dpi, VW5 induced a narrower set of host transcriptional changes than VW4, particularly in gene categories associated with feeding site initiation and host cell reprogramming. Importantly, because GO enrichment analysis was restricted to strain-specific gene sets, these differences do not indicate a complete absence of host manipulation by VW5 but rather suggest that VW5 has lost the ability to trigger a specific subset of host responses required for efficient feeding site establishment and maturation.

Based on these findings, we propose a trade-off model in which acquisition of *Mi-1* virulence involves loss or alteration of a nematode avirulence (*Avr*) gene product(s) that is required both for recognition by the *Mi-1* resistance pathway and for optimal host manipulation. Loss of such an *Avr* gene would enable evasion of *Mi-1*–mediated defense while simultaneously compromising feeding site establishment, nutrient acquisition, and reproduction on susceptible hosts. This hypothesis is consistent with previous work demonstrating that VW5 arose directly from VW4 under selection on *Mi-1* tomato and that the two strains are nearly genetically identical, differing primarily by the absence of the *Cg-1* cDNA fragment required for *Mi-1*–mediated resistance [17].

Although our results are consistent with a loss-of-*Avr* trade-off model, alternative evolutionary scenarios are also possible. Resistance breaking could, in some cases, arise through gain-of-function changes that enhance suppression of host immune signaling pathways rather than through loss of an *Avr* gene. For example, mutations that increase the efficacy or expression of effectors targeting defense-associated signaling networks could permit evasion of *Mi-1*–mediated recognition while maintaining or even enhancing host manipulation capacity. Under such a model, resistance breaking would not necessarily incur a fitness penalty and could explain the robust reproductive performance observed in some independently evolved field populations [14,34]. Distinguishing between loss-of-function and gain-of-function mechanisms will require functional characterization of candidate effectors and comparative genomic analyses of multiple virulent lineages.

From an applied perspective, these results underscore both the vulnerability and resilience of single-gene resistance strategies. Although Mi-1 has remained effective for decades, the emergence of resistance-breaking populations combined with environmental pressures such as increasing soil temperatures reveals limitations regarding durability. In addition, Mi-1 dosage may influence nematode performance, as reproduction of avirulent nematodes can in some cases be higher on heterozygous (Mi/mi) than homozygous (Mi/Mi) tomato varieties [34]. Because hybrid tomatoes are typically heterozygous for Mi-1, their widespread cultivation may create conditions that facilitate the accumulation of secondary mutations compensating for the fitness costs associated with virulence.

These findings also suggest that crop rotations could be designed to exploit the reduced performance of virulent strains on certain susceptible hosts. Such trade-offs may help slow the spread or eventual dominance of virulent genotypes in mixed cropping systems or rotation schemes incorporating susceptible cultivars. Determining how frequently these fitness costs occur in field populations will be essential for predicting the long-term durability of resistance. Root-knot nematodes belonging to *M. incognita* group (MIG) including *M. incognita*, *M. javanica*, *M. arenaria*, are polyploids and appear to retain substantial evolutionary flexibility despite obligate asexual reproduction. Recent genomic studies suggest that this flexibility may derive from its hybrid allopolyploid genome architecture, extensive post-polyploid chromosomal remodeling, and differential regulation of duplicated subgenomes [35–38]. Earlier work further indicates that gene conversion between divergent genome copies, together with transposable element activity, may provide additional sources of variation in the absence of meiosis [35,39]. Such genome plasticity likely contributes to the differences in host range and parasitic performance observed among isolates. Comparison of the genomes of VW4 and VW5 should therefore provide insights into the genetic mechanisms underlying this flexibility and, more specifically, into how virulence can evolve despite clonal reproduction.

Finally, the VW4-VW5 system provides a powerful framework for identifying nematode effectors and host susceptibility pathways that underpin successful parasitism. Genes and pathways identified through comparative genomic and transcriptomic analyses may serve as targets for next- generation control strategies, including susceptibility gene editing or diagnostic markers for early nematode infection. Together, our results demonstrate that resistance breaking in root-knot nematodes is not cost-free and that dissecting these trade-offs offers valuable insight into both the evolution of virulence and the development of durable nematode management strategies.

## Supporting information

S1 Fig A near isogenic population of *M. javanica* (VW5) overcomes resistance of *Mi-1* on tomato. (A) Resistant tomato (cv. Motelle) containing the *Mi-1* gene infected with *M. javanica* wildtype VW4 and resistance-breaking VW5. (B) The average numbers of eggs produced by VW5 and VW4 on cv. Motelle. Each data point represents an individual root system. Bars indicate means ± standard error of the mean (SEM) Statistical significance was assessed using an unpaired Student’s t-test (*p* < 0.05); significant difference is indicated by letters, (n = 30 roots per treatment).
(PDF)

S2 Fig Root fresh weights show a difference between roots infected with VW4 or VW5 for cucumber only. (A) Average tomato root weight, (B) Average cucumber root weight, (C) Average rice root weight. Fresh weight is recoded in grams. Each data point represents an individual root system. Bars indicate means ± standard error of the mean (SEM). Statistical significance was determined using unpaired Student’s t-tests, with p values shown; different letters indicate statistically significant differences (*p* < 0.05). Data are pooled from three independent biological experiments (tomato, n = 34; cucumber, n = 18; rice, n = 26).
(PDF)

S3 Fig Anatomy of uninfected roots of tomato and cucumber. Images of traversed sections of uninfected tomato roots of susceptible cv. Moneymaker (A and D), resistant cv. Motelle (B and E) and susceptible cucumber cv. Marketmore76 (C and F) at the late stage of primary (A-C) and the early stage of secondary root growth (D-F). Abbreviations: C, cortex; Ep, epidermis; Ex, exodermis; P, pericycle; Ph, phloem; RH, root hair; SX, secondary xylem vessel. Small yellow arrows indicate positions of tissues. Black arrows indicate the axis of primary xylem bundles. Asterisk indicates central metaxylem vessel. Scale bars: 50 µm.
(PDF)

S4 Fig Ultrastructure of giant cells developed in tomato roots at 7 dpi. Transmission electron microscopy (A - L) images of transverse sections of susceptible cv. Moneymaker (A, B, D, E, G, H, J, and K) and resistant cv. Motelle (C, F, I, and L) infected with avirulent VW4 strain (A, D, G, and J) or virulent VW5 strain (B, C, E, F, H, I, K, and L). Double tails arrows indicate plasmodesmata in thin fragments of cell walls between giant cells (D, E, and I). Scale bars: 2 µm. Abbreviations: CW, cell wall; ER, endoplasmic reticulum; GC, giant cell; M, mitochondrion; No, nucleolus; Nu, nucleus; P, parenchymatic cell; Pl, plastid; SE, sieve tube element; X, xylem vessel; V, vacuole.
(PDF)

S5 Fig Ultrastructure of giant cells developed in tomato roots at 28 dpi. Transmission electron microscopy images (A-L) of transverse sections of susceptible cv. Moneymaker (A, B, D, E, G, H, J, and K) and resistant cv. Motelle (C, F, I, and L) infected with avirulent VW4 strain (A, D, G, and J) or virulent VW5 strain (B, C, E, F, H, I, K, and L). Scale bars: 2 µm. Abbreviations: CW, cell wall; ER, endoplasmic reticulum; GC, giant cell; M, mitochondrion; No, nucleolus; Nu, nucleus; Pl, plastid; X, xylem vessel; V, vacuole.
(PDF)

S6 Fig Rplot representation of all significantly up- and downregulated genes compared to uninoculated roots at 7 and 28 dpi. Cucumber roots were inoculated with VW4 or VW5 strain of *M. javanica.* Galls were collected at 7 and 28 dpi. Gene significance was compared individually to the respective control roots. Significantly upregulated genes are shown in red and significantly downregulated genes are shown in blue. The number of genes is listed under and above each set. y-axis shows log2_foldchange.
(PDF)

S7 Fig Cucumber genes are more significantly and abundantly regulated during VW4 than VW5 infection. Volcano plots were generated using ggplot2 package in R Studio and shows genes expressed in cucumber during infection with either VW4 or VW5 at 7 and 28 dpi. (A) Significant gene expression after VW4 infection compared to the uninoculated roots at 7dpi. (B) Significant gene expression after VW5 infection compared to the uninoculated roots at 7 dpi. (C) Significant gene expression after VW4 infection compared to uninoculated roots at 28 dpi. (D) Significant gene expression after VW5 infection compared to uninoculated roots at 28 dpi. p < 0.05 for significance cut off. Significantly upregulated genes are shown in orange, while significantly downregulated genes are shown in blue, non-significant genes are grey (behind and under the dotted lines). The x-axis represents the log_2_(foldchange) and the y-axis represents the -log10(adjusted p-value).
(PDF)

S8 Fig Top 5 most significantly expressed up- and downregulated genes functions and domains. Top 5 most significant downregulated genes in blue. Top 5 most significant upregulated in orange. (A) Top 5 significant genes up- and downregulated for VW4 infection at 7 dpi, (B) Top 5 significant genes up- and downregulated for VW5 infection at 7 dpi, (C) Top 5 most significant genes up- and downregulated for VW4 infection at 28 dpi, and (D) Top 5 most significant genes up and downregulated for VW5 infection at 28 dpi. Gene number is listed along with associated Pfam predicted domain and function for each gene using InterPro data base.
(PDF)

S9 Fig GO-enrichment analysis of total cucumber genes up- and downregulated genes at 7 dpi. (A) Significantly upregulated gene families for VW4 infected roots at 7 dpi. (B) Significantly downregulated gene families for VW4 infected roots at 7 dpi. (C) Significantly upregulated gene families following VW5 infected roots at 7 dpi. (D) Significantly downregulated gene families following VW5 infected roots at 7 dpi. GO biological process terms are shown on the y-axis, and gene ratio is shown on the x-axis. Size of the circle indicates number of genes associated with each function. Circle color indicates p-value significance where -log10(adjusted p-value) and *p* < 0.05. Top 15 genes are displayed.
(PDF)

S10 Fig GO-enrichment analysis of total cucumber genes up- and downregulated genes at 28 dpi. (A) Significantly upregulated gene families for VW4 infected roots at 28 dpi. (B) Significantly downregulated gene families for VW4 infected roots at 28 dpi. (C) Significantly upregulated gene families following VW5 infected roots at 28 dpi. (D) Significantly downregulated gene families following VW5 infected roots at 28 dpi. GO biological process terms are shown on the y-axis, and gene ratio is shown on the x-axis. Circle size indicates number of genes associated with each function. Circle color indicated p-value significance where -log10(adjusted p-value) and *p* < 0.05. Top 15 genes are displayed.
(PDF)

S1 Table Summary of parameters measured comparing infection between VW4 and VW5 on susceptible tomato. (PDF)

S1 Data VW4 Differentially Expressed Genes (DEGs) 7 dpi. (XLSX)

S2 Data VW4 Differentially Expressed Genes (DEGs) 28 dpi. (XLSX)

S3 Data VW5 Differentially Expressed Genes (DEGs) 7 dpi. (XLSX)

S4 Data VW5 Differentially Expressed Genes (DEGs) 28 dpi. (XLSX)

S5 Data VW4 Uniquely Upregulated Genes 7 dpi. (XLSX)

S6 Data VW4 uniquely Downregulated genes 7 dpi. (XLSX)

S7 Data VW4 Uniquely Upregulated Genes 28 dpi. (XLSX)

S8 Data VW4 uniquely Downregulated genes 28 dpi. (XLSX)

S9 Data VW5 Uniquely Upregulated Genes 7 dpi. (XLSX)

S10 Data VW5 uniquely Downregulated genes 7 dpi. (XLSX)

S11 Data VW5 Uniquely Upregulated Genes 28 dpi. (XLSX)

S12 Data VW5 uniquely Downregulated genes 28 dpi. (XLSX)
