# Supplementary material for: Resistance breaking Root-knot nematodes carry a fitness cost associated with defective feeding site development
Source: PLoS Pathog. 2026 Sep 11;22(9):e1014581. doi: 10.1371/journal.ppat.1014581 (PMC13568518; doi:10.1371/journal.ppat.1014581)
Supplement: Supplementary file 1 [file ppat.1014581.s001.pdf]

Supplemental Figures

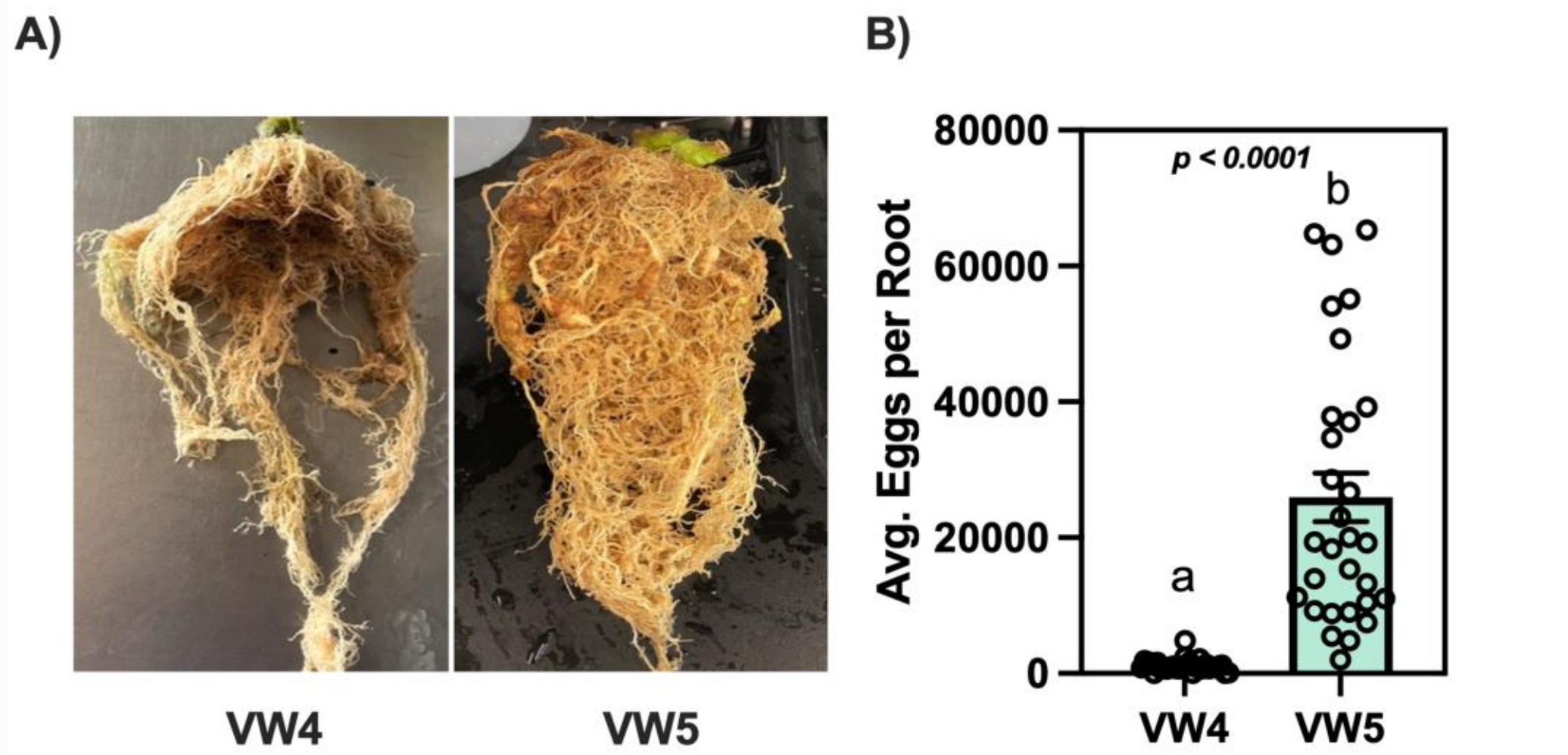

S1 Fig: A near isogenic population of *M. javanica* (VW5) overcomes resistance of *Mi-1* on tomato. (A) Resistant tomato (cv. Motelle) containing the *Mi-1* gene infected with *M. javanica* wildtype VW4 and resistance-breaking VW5. (B) The average numbers of eggs produced by VW5 and VW4 on cv. Motelle. Each data point represents an individual root system. Bars indicate means ± standard error of the mean (SEM) Statistical significance was assessed using an unpaired Student's t-test ($p < 0.05$); significant difference is indicated by letters, (n = 30 roots per treatment).
