# Supplementary material for: Resistance breaking Root-knot nematodes carry a fitness cost associated with defective feeding site development
Source: PLoS Pathog. 2026 Sep 11;22(9):e1014581. doi: 10.1371/journal.ppat.1014581 (PMC13568518; doi:10.1371/journal.ppat.1014581)
Supplement: Supplementary file 2 [file ppat.1014581.s002.pdf]

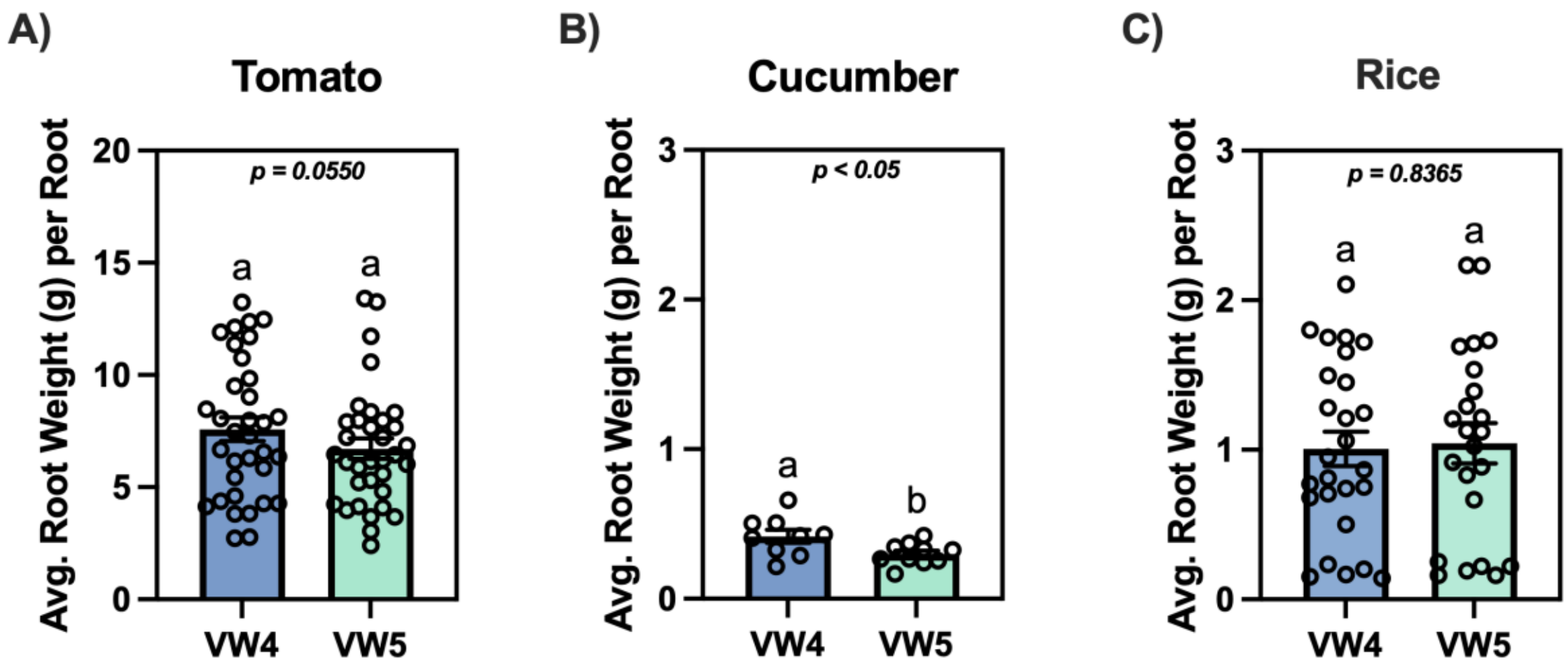

S2 Fig: Root fresh weights show a difference between roots infected with VW4 or VW5 for cucumber only. (A) Average tomato root weight, (B) Average cucumber root weight, (C) Average rice root weight. Fresh weight is recoded in grams. Each data point represents an individual root system. Bars indicate means ± standard error of the mean (SEM). Statistical significance was determined using unpaired Student's t-tests, with p values shown; different letters indicate statistically significant differences ($p < 0.05$). Data are pooled from three independent biological experiments (tomato, n = 34; cucumber, n = 18; rice, n = 26).
