# Supplementary material for: Resistance breaking Root-knot nematodes carry a fitness cost associated with defective feeding site development
Source: PLoS Pathog. 2026 Sep 11;22(9):e1014581. doi: 10.1371/journal.ppat.1014581 (PMC13568518; doi:10.1371/journal.ppat.1014581)
Supplement: Supplementary file 3 [file ppat.1014581.s003.pdf]

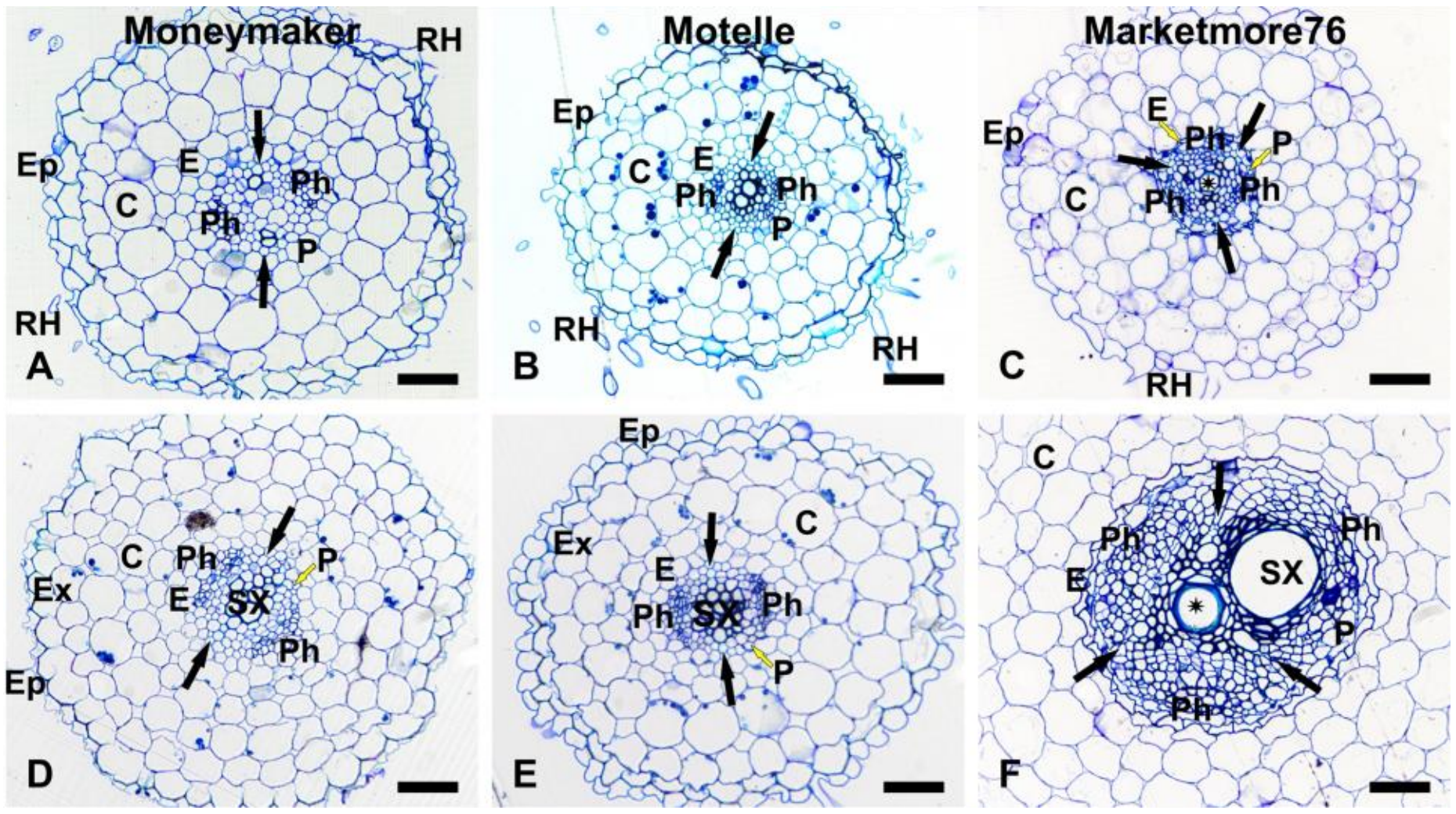

S3 Fig: Anatomy of uninfected roots of tomato and cucumber. Images of traversed sections of uninfected tomato roots of susceptible cv. Moneymaker (A and D), resistant cv. Motelle (B and E) and susceptible cucumber cv. Marketmore76 (C and F) at the late stage of primary (A-C) and the early stage of secondary root growth (D-F). Abbreviations: C, cortex; Ep, epidermis; Ex, exodermis; P, pericycle; Ph, phloem; RH, root hair; SX, secondary xylem vessel. Small yellow arrows indicate positions of tissues. Black arrows indicate the axis of primary xylem bundles. Asterisk indicates central metaxylem vessel. Scale bars: 50 µm.
