# Supplementary material for: Resistance breaking Root-knot nematodes carry a fitness cost associated with defective feeding site development
Source: PLoS Pathog. 2026 Sep 11;22(9):e1014581. doi: 10.1371/journal.ppat.1014581 (PMC13568518; doi:10.1371/journal.ppat.1014581)
Supplement: Supplementary file 4 [file ppat.1014581.s004.pdf]

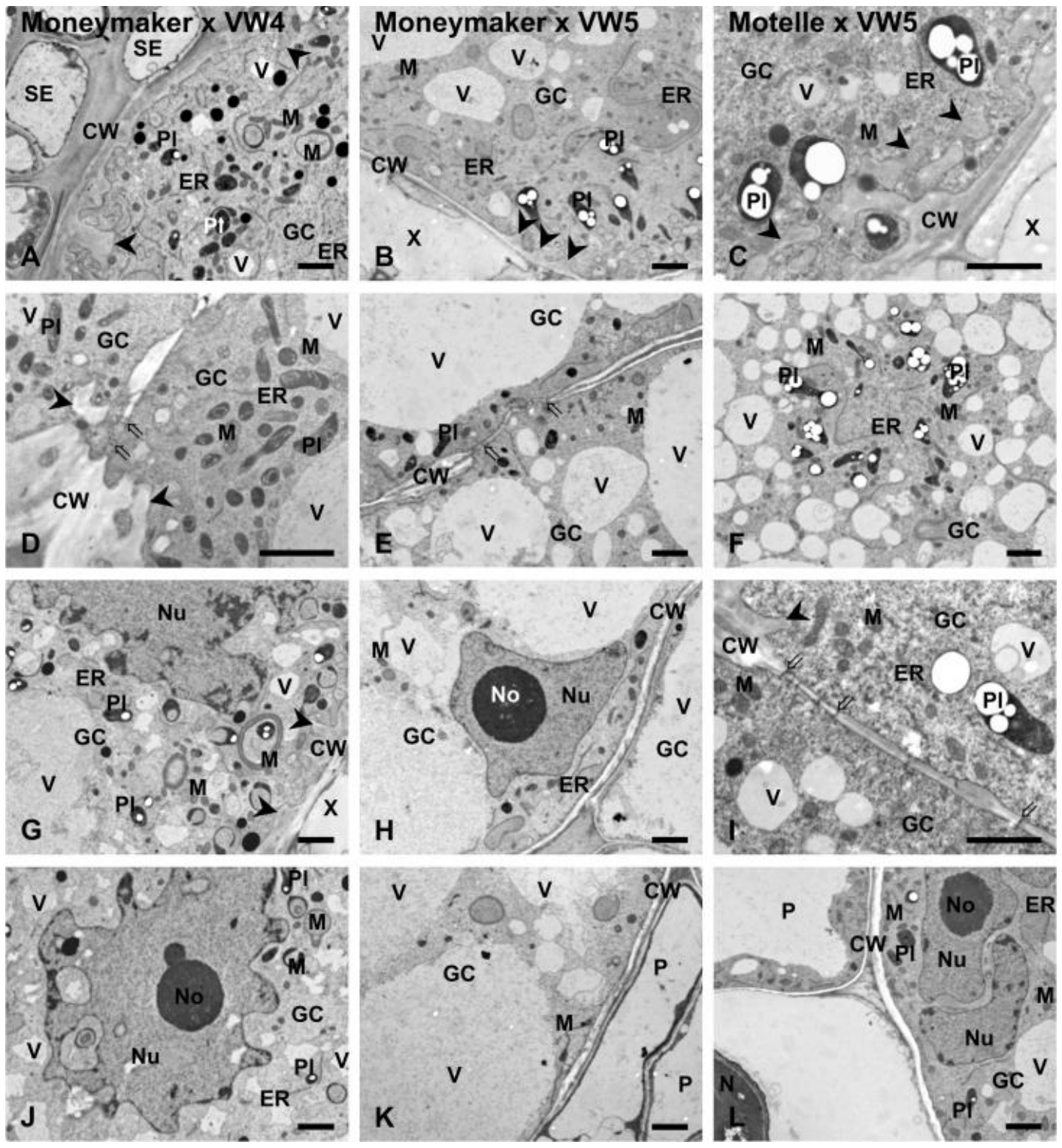

S4 Fig: Ultrastructure of giant cells developed in tomato roots at 7 dpi. Transmission electron microscopy (A - L) images of transverse sections of susceptible cv. Moneymaker (A, B, D, E, G, H, J, and K) and resistant cv. Motelle (C, F, I, and L) infected with avirulent VW4 strain (A, D, G, and J) or virulent VW5 strain (B, C, E, F, H, I, K, and L). Double tails arrows indicate plasmodesmata in thin fragments of cell walls between giant cells (D, E, and I). Scale bars: 2 µm. Abbreviations: CW, cell wall; ER, endoplasmic reticulum; GC, giant cell; M, mitochondrion; No, nucleolus; Nu, nucleus; P, parenchymatic cell; Pl, plastid; SE, sieve tube element; X, xylem vessel; V, vacuole.
