# Supplementary material for: Resistance breaking Root-knot nematodes carry a fitness cost associated with defective feeding site development
Source: PLoS Pathog. 2026 Sep 11;22(9):e1014581. doi: 10.1371/journal.ppat.1014581 (PMC13568518; doi:10.1371/journal.ppat.1014581)
Supplement: Supplementary file 5 [file ppat.1014581.s005.pdf]

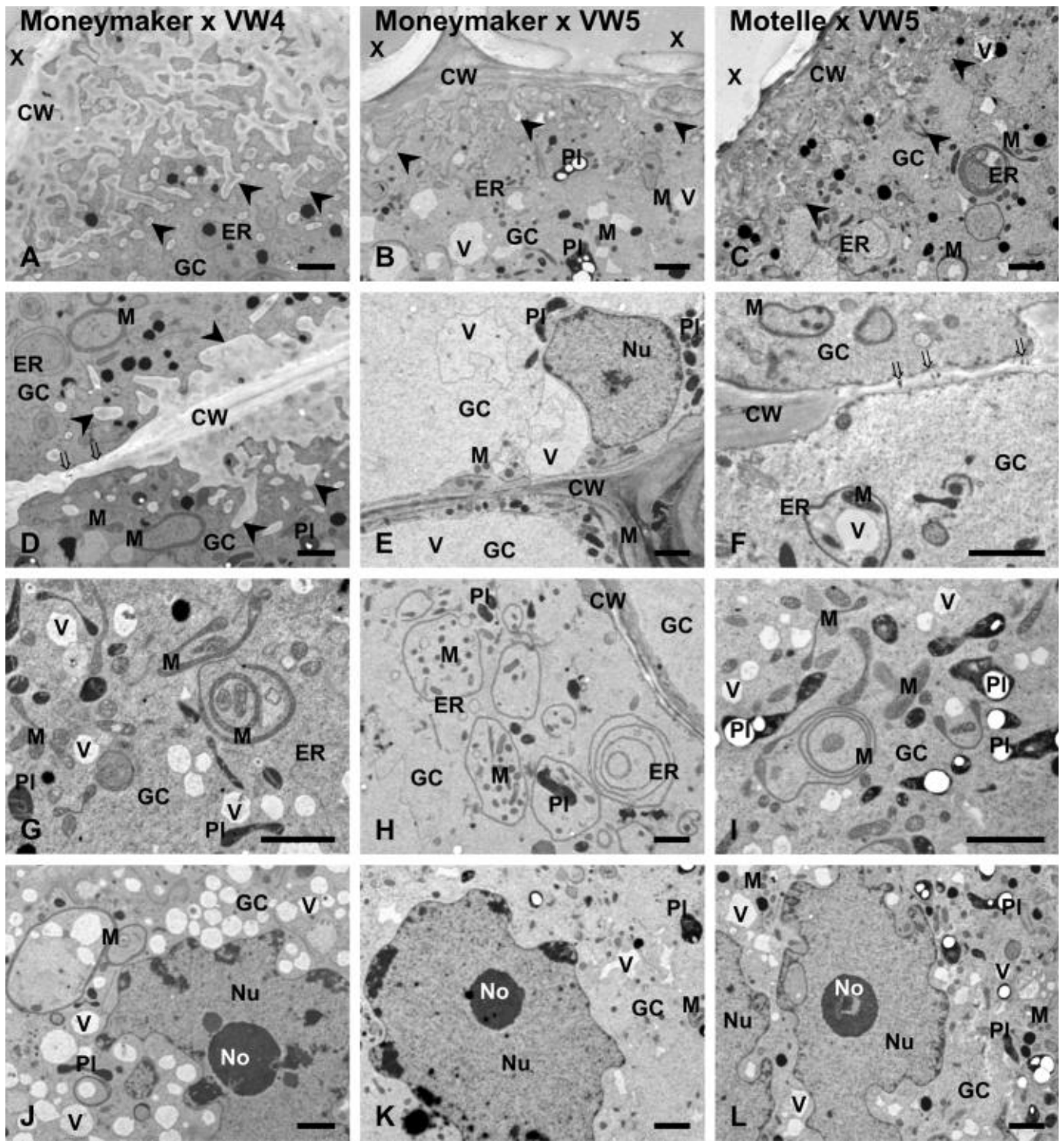

S5 Fig: Ultrastructure of giant cells developed in tomato roots at 28 dpi. Transmission electron microscopy images (A-L) of transverse sections of susceptible cv. Moneymaker (A, B, D, E, G, H, J, and K) and resistant cv. Motelle (C, F, I, and L) infected with avirulent VW4 strain (A, D, G, and J) or virulent VW5 strain (B, C, E, F, H, I, K, and L). Scale bars: 2 μm. Abbreviations: CW, cell wall; ER, endoplasmic reticulum; GC, giant cell; M, mitochondrion; No, nucleolus; Nu, nucleus; Pl, plastid; X, xylem vessel; V, vacuole.
