# Supplementary material for: Resistance breaking Root-knot nematodes carry a fitness cost associated with defective feeding site development
Source: PLoS Pathog. 2026 Sep 11;22(9):e1014581. doi: 10.1371/journal.ppat.1014581 (PMC13568518; doi:10.1371/journal.ppat.1014581)
Supplement: Supplementary file 6 [file ppat.1014581.s006.pdf]

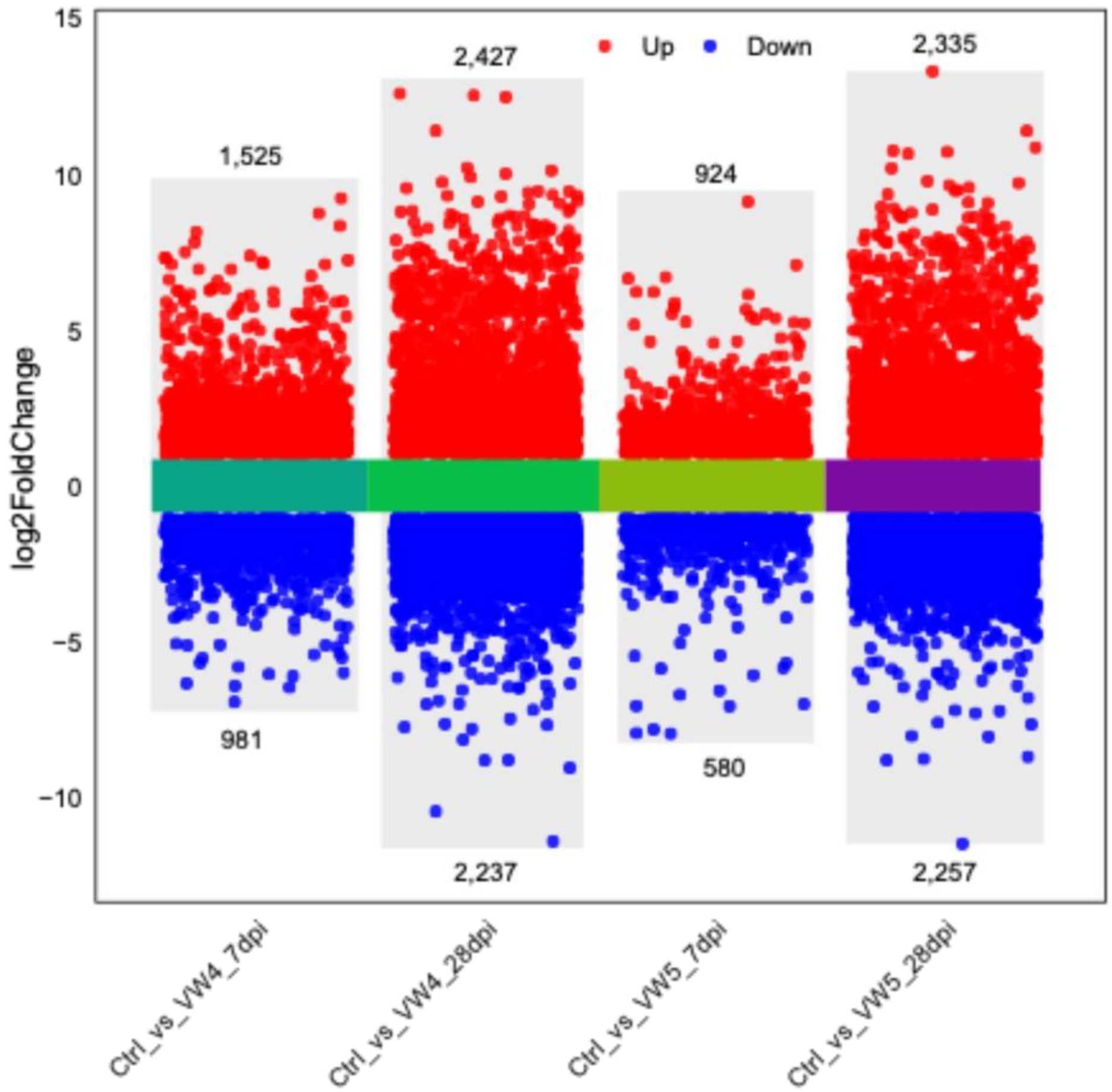

S6 Fig: Rplot representation of all significantly up- and downregulated genes compared to uninoculated roots at 7 and 28 dpi. Cucumber roots were inoculated with VW4 or VW5 strain of *M. javanica.* Galls were collected at 7 and 28 dpi. Gene significance was compared individually to the respective control roots. Significantly upregulated genes are shown in red and significantly downregulated genes are shown in blue. The number of genes is listed under and above each set. y-axis shows log2_foldchange.
