# Supplementary material for: Resistance breaking Root-knot nematodes carry a fitness cost associated with defective feeding site development
Source: PLoS Pathog. 2026 Sep 11;22(9):e1014581. doi: 10.1371/journal.ppat.1014581 (PMC13568518; doi:10.1371/journal.ppat.1014581)
Supplement: Supplementary file 7 [file ppat.1014581.s007.pdf]

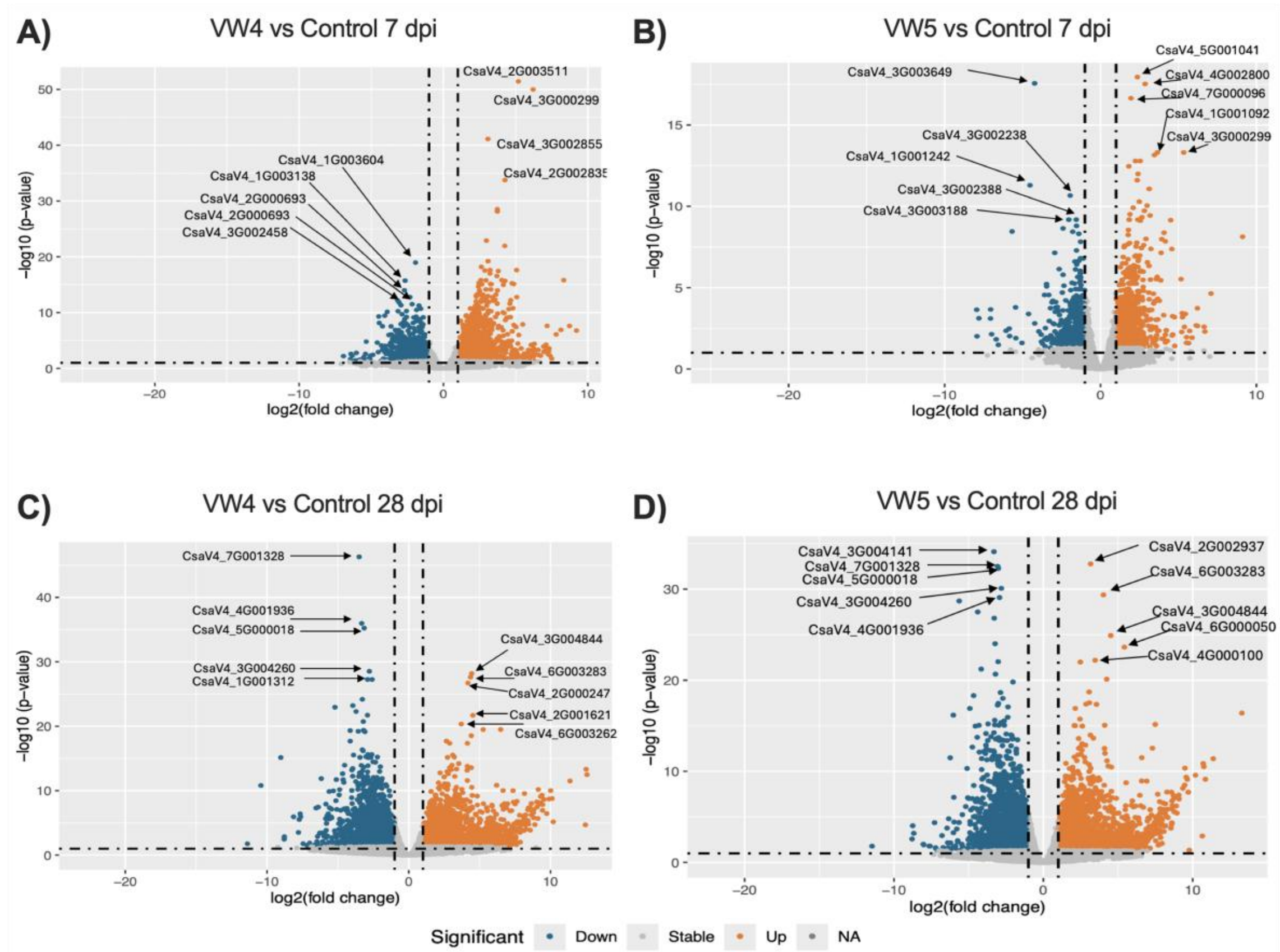

S7 Fig: Cucumber genes are more significantly and abundantly regulated during VW4 than VW5 infection. Volcano plots were generated using ggplot2 package in R Studio and shows genes expressed in cucumber during infection with either VW4 or VW5 at 7 and 28 dpi. (A) Significant gene expression after VW4 infection compared to the uninoculated roots at 7dpi. (B) Significant gene expression after VW5 infection compared to the uninoculated roots at 7 dpi. (C) Significant gene expression after VW4 infection compared to uninoculated roots at 28 dpi. (D) Significant gene expression after VW5 infection compared to uninoculated roots at 28 dpi. $p < 0.05$ for significance cut off. Significantly upregulated genes are shown in orange, while significantly downregulated genes are shown in blue, non-significant genes are grey (behind and under the dotted lines). The x-axis represents the $\log_2$(foldchange) and the y-axis represents the -log10(adjusted p-value).
