# Supplementary material for: Resistance breaking Root-knot nematodes carry a fitness cost associated with defective feeding site development
Source: PLoS Pathog. 2026 Sep 11;22(9):e1014581. doi: 10.1371/journal.ppat.1014581 (PMC13568518; doi:10.1371/journal.ppat.1014581)
Supplement: Supplementary file 8 [file ppat.1014581.s008.pdf]

A) VW4 vs Control 7 dpi

Top 5 Down Regulated Genes	Pfam Data Base	Top 5 Up Regulated Genes	Pfam Data Base
CsaV4_1G003604	PF01513 = ATP-NAD kinase N-terminal domain	CsaV4_2G003511	PF01397 = Terpene synthase, N-terminal domain
	PF20143 = ATP-NAD kinase C-terminal domain		PF03936 = Terpene synthase family, metal binding domain
CsaV4_1G003138	PF07887 = Calmodulin binding protein-like N-terminal domain	CsaV4_3G000299	PF14547 = Hydrophobic seed protein
	PF20451 = Calmodulin binding protein central domain		
CsaV4_2G000693	PF00067 = Cytochrome P450	CsaV4_3G002855	PF03000 = NPH3 family
			PF00651 = BTB/POZ domain
CsaV4_5G003453	PF01582 = TIR Domain	CsaV4_2G002835	PF00892 = EamA-like transporter family
	PF00931 = NB-ARC domain		
	PF13855 = Leucine rich repeat		
	PF20160 = C-JID domain		
CsaV4_3G002458	Unknown	CsaV4_6G003854	PF01190 = Pollen protein Ole e 1 like

B) VW5 vs Control 7 dpi

Top 5 Down Regulated Genes	Pfam Data Base	Top 5 Up Regulated Genes	Pfam Data Base
CsaV4_3G003649	PF01161 = Phosphatidylethanolamine-binding protein	CsaV4_5G001041	Unknown
CsaV4_3G002238	PF00407 = Pathogenesis-related protein Bet v 1 family	CsaV4_4G002800	PF00202 = Aminotransferase class-III
CsaV4_1G001242	PF11883 = Unknown	CsaV4_7G000096	PF01190 = Pollen protein Ole e 1 like
	PF07714 = Protein tyrosine and serine/threonine kinase		
	PF08276 = PAN-like domain		
	PF00954 = S-locus glycoprotein domain		PF00160 = Cyclophilin type peptidyl-prolyl cis-trans isomerase/CLD
	PF01453 = D-mannose binding lectin		
CsaV4_3G002388	Unknown	CsaV4_1G001092	PF13962 = Unknown
			PF12796 = Ankyrin repeats (3 copies)
CsaV4_3G003188	PF01370 = NAD dependent epimerase/dehydratase family	CsaV4_3G000299	Unknown
	PF13460 = NAD(P)H-binding		

C) VW4 vs Control 28 dpi

Top 5 Down Regulated Genes	Pfam Data Base	Top 5 Up Regulated Genes	Pfam Data Base
CsaV4_3G003649	PF01161 = Phosphatidylethanolamine-binding protein	CsaV4_5G001041	Unknown
CsaV4_3G002238	PF00407 = Pathogenesis-related protein Bet v 1 family	CsaV4_4G002800	PF00202 = Aminotransferase class-III
CsaV4_1G001242	PF11883 = Unknown	CsaV4_7G000096	PF01190 = Pollen protein Ole e 1 like
	PF07714 = Protein tyrosine and serine/threonine kinase		
	PF08276 = PAN-like domain		
	PF00954 = S-locus glycoprotein domain		PF00160 = Cyclophilin type peptidyl-prolyl cis-trans isomerase/CLD
	PF01453 = D-mannose binding lectin		
CsaV4_3G002388	Unknown	CsaV4_1G001092	PF13962 = Unknown
			PF12796 = Ankyrin repeats (3 copies)
CsaV4_3G003188	PF01370 = NAD dependent epimerase/dehydratase family	CsaV4_3G000299	Unknown
	PF13460 = NAD(P)H-binding		

D) VW5 vs Control 28 dpi

Top 5 Down Regulated Genes	Pfam Data Base	Top 5 Up Regulated Genes	Pfam Data Base
CsaV4_3G004141	PF22932 = DUF569 associated ubiquitin-like domain	CsaV4_2G002937	PF14683 = Polysaccharide lyase family 4, domain III
	PF04601 = Beta-trefoil domain of unknown function (DUF569)		PF14686 = Polysaccharide lyase family 4, domain II
			PF06045 = Rhamnogalacturonate lyase family
CsaV4_7G001328	Unknown	CsaV4_6G003283	PF00560 = Leucine Rich Repeat
			PF13855 = Leucine rich repeat
			PF08263 = Leucine rich repeat N-terminal domain
			PF07714 = Protein tyrosine and serine/threonine kinase
CsaV4_5G000018	PF00069 = Protein kinase domain	CsaV4_3G004844	PF00069 = Protein kinase domain
			PF00560 = Leucine Rich Repeat
			PF13855 = Leucine rich repeat
			PF08263 = Leucine rich repeat N-terminal domain
CsaV4_3G004260	PF07002 = Copine	CsaV4_6G000050	PF03479 = Plants and Prokaryotes Conserved (PCC) domain
	PF13920 = Zinc finger, C3HC4 type (RING finger)		
	PF13041 = PPR repeat family		
	PF13812 = Pentatricopeptide repeat domain		
CsaV4_4G001936	Unknown	CsaV4_4G000100	PF00534 = Glycosyl transferases group 1
			PF00862 = Sucrose synthase first GT-B domain

S8 Fig: Top 5 most significantly expressed up- and downregulated genes functions and domains. Top 5 most significant downregulated genes in blue. Top 5 most significant upregulated in orange. (A) Top 5 significant genes up- and downregulated for VW4 infection at 7 dpi, (B) Top 5 significant genes up- and downregulated for VW5 infection at 7 dpi, (C) Top 5 most significant genes up- and downregulated for VW4 infection at 28 dpi, and (D) Top 5 most significant genes up and downregulated for VW5 infection at 28 dpi. Gene number is listed along with associated Pfam predicted domain and function for each gene using InterPro data base.
