# Supplementary material for: Resistance breaking Root-knot nematodes carry a fitness cost associated with defective feeding site development
Source: PLoS Pathog. 2026 Sep 11;22(9):e1014581. doi: 10.1371/journal.ppat.1014581 (PMC13568518; doi:10.1371/journal.ppat.1014581)
Supplement: Supplementary file 9 [file ppat.1014581.s009.pdf]

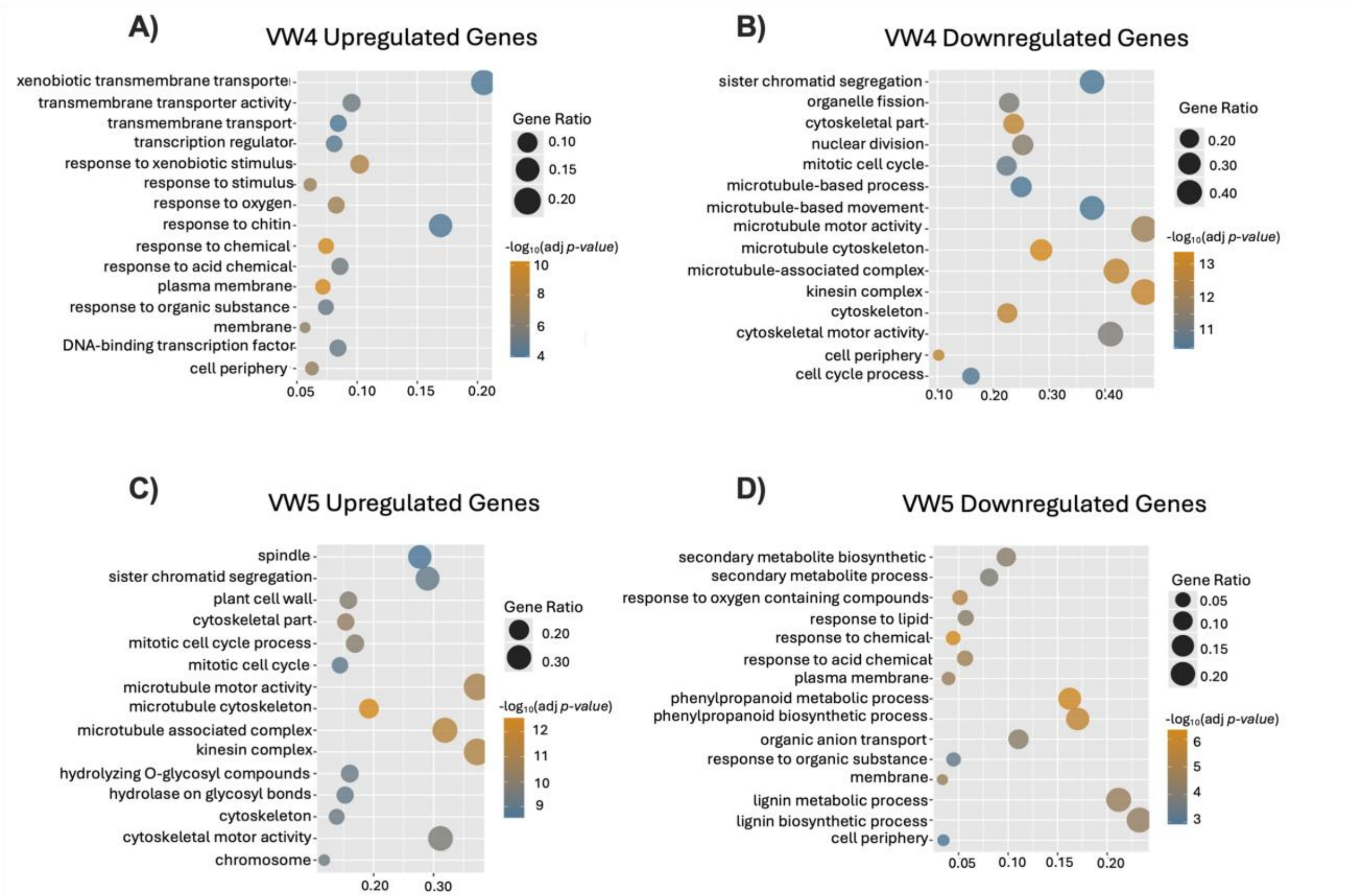

S9 Fig: **GO-enrichment analysis of total cucumber genes up- and downregulated genes at 7 dpi.** (A) Significantly upregulated gene families for VW4 infected roots at 7 dpi. (B) Significantly downregulated gene families for VW4 infected roots at 7 dpi. (C) Significantly upregulated gene families following VW5 infected roots at 7 dpi. (D) Significantly downregulated gene families following VW5 infected roots at 7 dpi. GO biological process terms are shown on the y-axis, and gene ratio is shown on the x-axis. Size of the circle indicates number of genes associated with each function. Circle color indicates p-value significance where -log10(adjusted p-value) and $p < 0.05$. Top 15 genes are displayed.
