# Supplementary material for: Resistance breaking Root-knot nematodes carry a fitness cost associated with defective feeding site development
Source: PLoS Pathog. 2026 Sep 11;22(9):e1014581. doi: 10.1371/journal.ppat.1014581 (PMC13568518; doi:10.1371/journal.ppat.1014581)
Supplement: Supplementary file 10 [file ppat.1014581.s010.pdf]

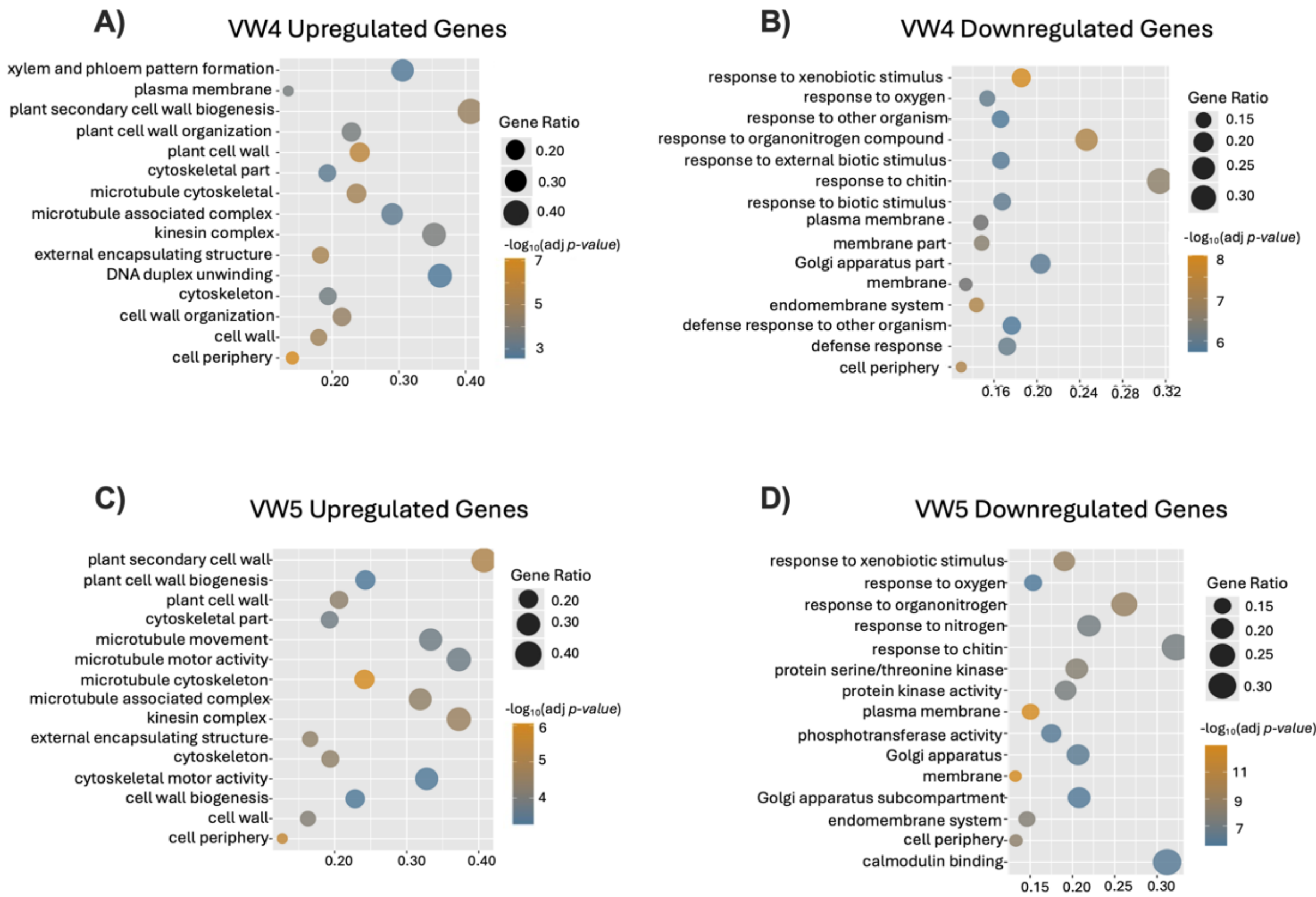

S10 Fig: GO-enrichment analysis of total cucumber genes up- and downregulated genes at 28 dpi. (A) Significantly upregulated gene families for VW4 infected roots at 28 dpi. (B) Significantly downregulated gene families for VW4 infected roots at 28 dpi. (C) Significantly upregulated gene families following VW5 infected roots at 28 dpi. (D) Significantly downregulated gene families following VW5 infected roots at 28 dpi. GO biological process terms are shown on the y-axis, and gene ratio is shown on the x-axis. Circle size indicates number of genes associated with each function. Circle color indicated p-value significance where -log10(adjusted p-value) and $p < 0.05$. Top 15 genes are displayed.
