# Supplementary material for: Resistance breaking Root-knot nematodes carry a fitness cost associated with defective feeding site development
Source: PLoS Pathog. 2026 Sep 11;22(9):e1014581. doi: 10.1371/journal.ppat.1014581 (PMC13568518; doi:10.1371/journal.ppat.1014581)
Supplement: Supplementary file 11 [file ppat.1014581.s011.pdf]

Supplemental Table 1: Summary of parameters measured comparing infection between VW4 and VW5 on susceptible tomato. Parameters and separate experiments are depicted. Values listed as lower for VW5 are significantly lower ($p < 0.05$).

Parameter	Experiment 1	Experiment 2	Experiment 3
Root weight	Not Significant	Not Significant	Not Significant
Egg Production	**Lower for VW5**	**Lower for VW5**	**Lower for VW5**
Number of Galls	Not Significant	**Lower for VW5**	Not Significant
Number of J2s	Not Significant	Not Significant	Not Significant
Number of Females	Lower for VW4	Lower for VW5	Not Significant
Total Population	Lower for VW4	Lower for VW5	Not Significant
Eggs per Female	**Lower for VW5**	Not Significant	**Lower for VW5**
